# Meta-analysis of levamisole absorption and disposition across diverse species using a minimal physiologically-based pharmacokinetic model

**DOI:** 10.1007/s40005-025-00770-6

**Published:** 2025-09-11

**Authors:** ChunFu Cheng, Yoo-Seong Jeong, William J. Jusko

**Affiliations:** 1 Department of Pharmaceutical Sciences, School of Pharmacy and Pharmaceutical Sciences, State University of New York at Buffalo, Buffalo, NY USA; 2 Division of Pharmacokinetics, Pharmacodynamics, and Systems Pharmacology, Department of Pharmaceutical Sciences, School of Pharmacy and Pharmaceutical Sciences, State University of New York at Buffalo, 404 Pharmacy Building, Buffalo, NY14214-803 USA

**Keywords:** Levamisole, Clearance, Interspecies scaling, Physiological pharmacokinetics

## Abstract

**Purpose:**

Pharmacokinetic (PK) data for levamisole, an important immunostimulant and antiparasitic agent, were identified in 18 species providing sufficient PK data following oral (PO) and/or intravenous (IV) administration for assessment and comparison.

**Methods:**

Pharmacokinetic parameters were sought in all species for traditional allometric assessment. Among these, 2 bird and 6 mammalian species provided sufficient data for joint modeling using traditional compartmental PK and minimal physiologically-based pharmacokinetic (mPBPK) methods.

**Results:**

Simple allometric scaling was first used examine clearance (*CL*), steady-state volume of distribution (*V*_*ss*_), absorption rate constant (*k*_*a*_), and bioavailability (*F*) in relation to body weight (*BW*) across species. The *V*_*ss*_ correlated well with *BW (Parameter = α·BW*^*b*^*)* with *b* = 0.89 (R^2^ = 0.81) whereas *CL* (*b* = 0.26, R^2^ = 0.46), *k*_*a*_ (*b* = 0.25, R^2^ = 0.14), and *F* (*b* = 0.08, R^2^ = 0.70) showed weaker correlations with ducks appearing as outliers for *CL*. Biexponential PK profiles were adequately captured using an allometric two-compartment model (2CM). Joint fitting of IV PK data from 8 species to a generalized mPBPK model, incorporating unified distribution parameters (e.g., tissue partition coefficient *K*_*p*_ and fractional distribution parameter *f*_*d*_), yielded good performance across species. The mPBPK model assuming high tissue permeability and species-specific *K*_*p*_ values for pig and chicken (*K*_*p, pig*_ and *K*_*p, chicken*_) best described the observed profiles. Oral bioavailability(*F*) was highly consistent across all species (50–80%), with the exception of goats.

**Conclusion:**

This study demonstrates that levamisole with rapid absorption and extensive metabolism exhibits largely consistent PK properties across species. Minimal PBPK modeling offers advantageous comparison of interspecies determinants of levamisole PK.

**Supplementary Information:**

The online version contains supplementary material available at 10.1007/s40005-025-00770-6.

## Introduction

Levamisole (LVM) exhibits diverse pharmacological effects, including anti-helminthic, anti-inflammatory, antioxidant, anti-neoplastic, and immunomodulatory actions (Chandy et al. 2016). The U.S. Food and Drug Administration (FDA) approved this drug in the 1980s for the treatment of rheumatoid arthritis and in 1990 as an adjuvant therapy for colon cancer owing to its anti-neoplastic properties (Amery and Bruynseels 1992). More recently, its immunological effects have drawn attention for their potential in enhancing the human immune response to COVID-19 and alleviating symptoms such as cough and shortness of breath in non-hospitalized COVID-19 patients (Roostaei Firozabad et al. 2021). However, LVM is associated with serious adverse effects, including vasculitis, multifocal inflammatory leukoencephalopathy, agranulocytosis, and nervous system disorders (Campillo et al. 2022). Toxicological studies are warranted to further evaluate its potential risk for inducing cytokine storms (Al-Kuraishy et al. 2021). In addition, LVM is illicitly used as a cocaine adulterant, resulting in the production of hazardous metabolites that cause severe dermatological side effects, presenting a significant public health challenge. These findings underscore the pleiotropic nature of LVM, encompassing both therapeutic benefits and significant risks.

LVM, the active S(-) isomer of tetramisole, is a basic compound with a pKa value of 6.98 and a molecular weight of 204.292 g/mol. It demonstrates high aqueous solubility (210 mg/mL) and moderate lipophilicity (log *P* = 1.84). Although limited evidence for its permeability is available in the literature (Kambayashi et al. 2023), LVM exhibited permeability coefficients higher than those of a high-permeability reference metoprolol in both in vitro cell monolayer and in situ intestinal perfusion studies (Xiangxiu et al. 2020). The pharmacokinetics (PK) of LVM exhibit some unique characteristics across species: The PK of LVM in multiple species is described by biexponential disposition kinetics, while a mono-exponential decline was observed in dogs and humans (Luyckx et al. 1982). In man, LVM demonstrates rapid absorption, with the time to reach maximum plasma concentration ($\:{T}_{max}$) of approximately 1.5 h. The terminal-phase half-life of LVM is approximately 5.6 h in man, whereas the value ranges from 1 h in rats and 4 h in cows (Graziani and De Martin 1977). The total clearance ($\:C{L}_{tot}$) in man is reported as 33.8 L/h, with renal clearance accounting for only 1.8 L/h, indicating that non-renal pathways, likely hepatic metabolism, contribute substantially to overall clearance. The volume of distribution ($\:{V}_{d}$) was observed as 266 L in man (Kouassi et al. 1986), and tissue distribution studies across species shows limited partitioning of LVM into muscle, fat, and brain tissues (Graziani and De Martin 1977).

Although various PK properties of LVM have been reported, a comprehensive PK comparison across multiple species remains lacking. Allometric scaling is a widely used method for evaluating PK across species and predicting clinical PK from animal data (Jansen et al. 2020). This approach becomes more useful when assessed with modeling techniques. For example, PK data from multiple species have been analyzed for ketoprofen and dexamethasone, where the plasma PK profiles were jointly described using a two-compartment model (2CM) with sets of parameters derived from allometry (Lepist and Jusko 2004; Song and Jusko 2021). Across-species PK data for metformin and dexamethasone were modeled utilizing a minimal physiologically-based pharmacokinetic (mPBPK) framework (Jeong and Jusko 2021; Song and Jusko 2021). This approach, which simplifies traditional PBPK models by focusing on key physiological characteristics and utilizing plasma PK data alone (Cao and Jusko 2012), has enabled the generation of kinetic parameters for global application across species.

This report comprehensively evaluates the PK properties of LVM across multiple species. A literature search and meta-analysis were conducted to gather available PK data for LVM. Intravenous (IV) and oral (PO) PK data from 8 selected species could be analyzed using 2CM and mPBPK models to identify conserved PK properties across species. This consolidated and quantitative assessment of the PK characteristics of LVM across multiple species offers unique insights into its interspecies PK.

## Materials and methods

### Research methods and steps

**Fig. 1:**
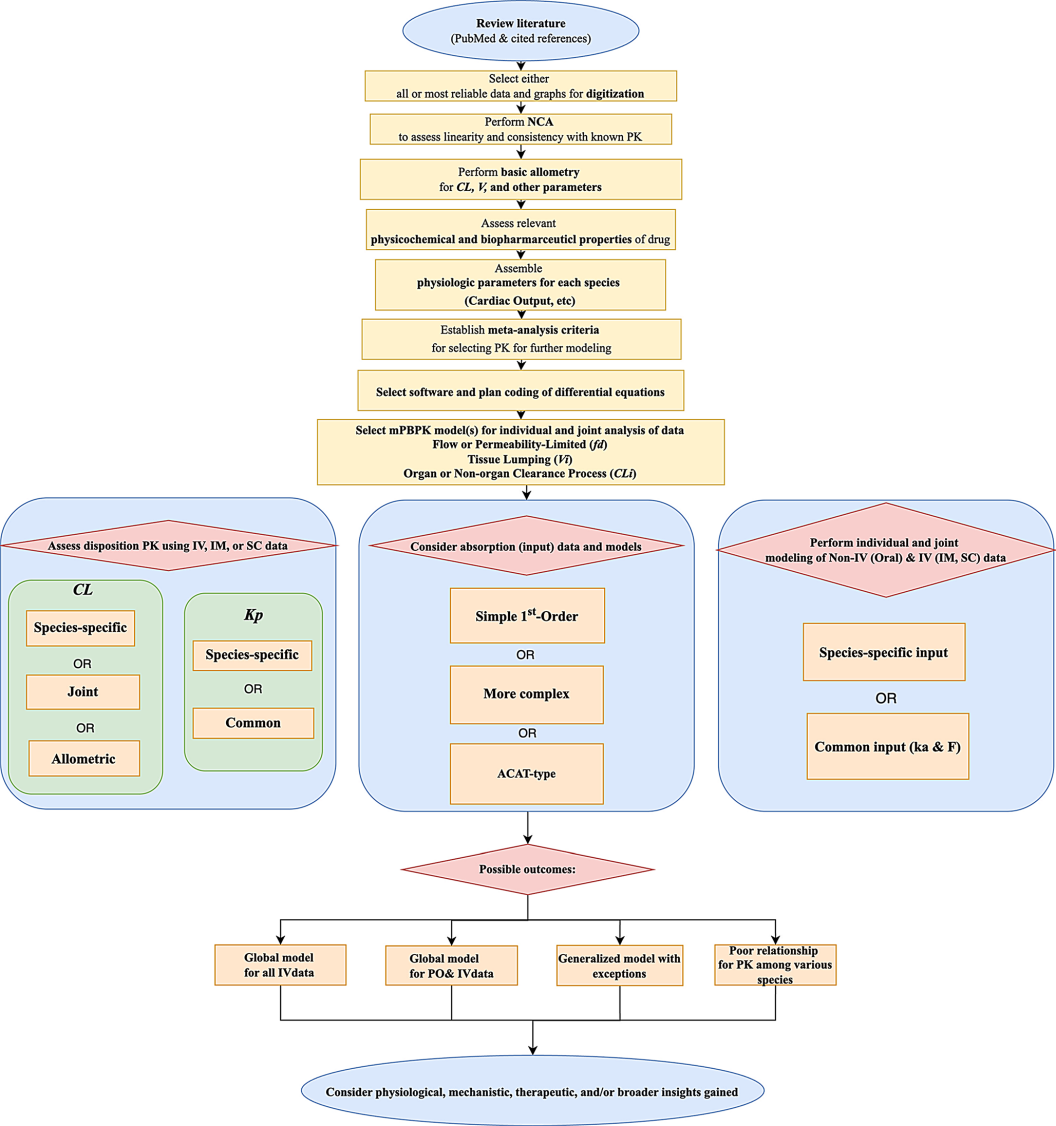
Flow chart for meta-analysis of mPBPK among species

The research methodology involved meta-analyses and integrated PK model evaluations. A comprehensive flowchart that outlines each step, from initial data collection to the final conceptual insights gained from the analysis, is shown in Fig. 1.

**Table 1:** Comparison of clearance ($\:CL$, L/h/kg) values for LVM from various sources and methods

Species ($\:\boldsymbol{B}\boldsymbol{W}$, kg)	Reported CL	NCA^a^ CL	2CM^b^ CL	2CM^c^ CL	Joint 2CM^d^ CL	mPBPK individual CL^b^	mPBPK individual CL^c^	Joint mPBPK CL^d^
Duck (2.5)	0.28	0.24	0.25	0.21	0.20 (7.54)	0.24	0.24	0.216 (4.25)
Rabbit (3)	2.89	2.95	2.64	2.71	1.09 (7.79)	2.22	2.21	1.46 (6.37)
Chicken (4.5)	2.46	2.36	2.43	2.42	0.93 (9.07)	2.03	2.02	2.59 (4.05)
Goat (18)	0.58	0.36	0.37	0.37	0.38 (0.90)	0.42	0.42	0.359 (1.05)
Dog (20.7)	0.54	0.55	0.57	0.57	0.49 (4.39)	0.57	0.54	0.464 (4.12)
Sheep (26)	1.16	1.13	1.12	1.09	0.99 (5.88)	1.00	0.98	0.994 (4.56)
Pig (39.2)	0.39	0.42	0.42	0.44	0.27 (7.43)	0.39	0.39	0.371 (7.28)
Human (70)	0.37	0.50	0.24	NAe	0.25 (3.72)	0.228	NA	0.249 (5.52)

^a^ Non-compartmental analysis (NCA) conducted using Phoenix WinNonlin

^b^ Fitting of only IV data for each individual species

^c^ Fitting of IV and oral data combined for each individual species

^d^ Joint fitting of only IV data across species; values in parentheses are CV% of estimates

**Fig. 2:**
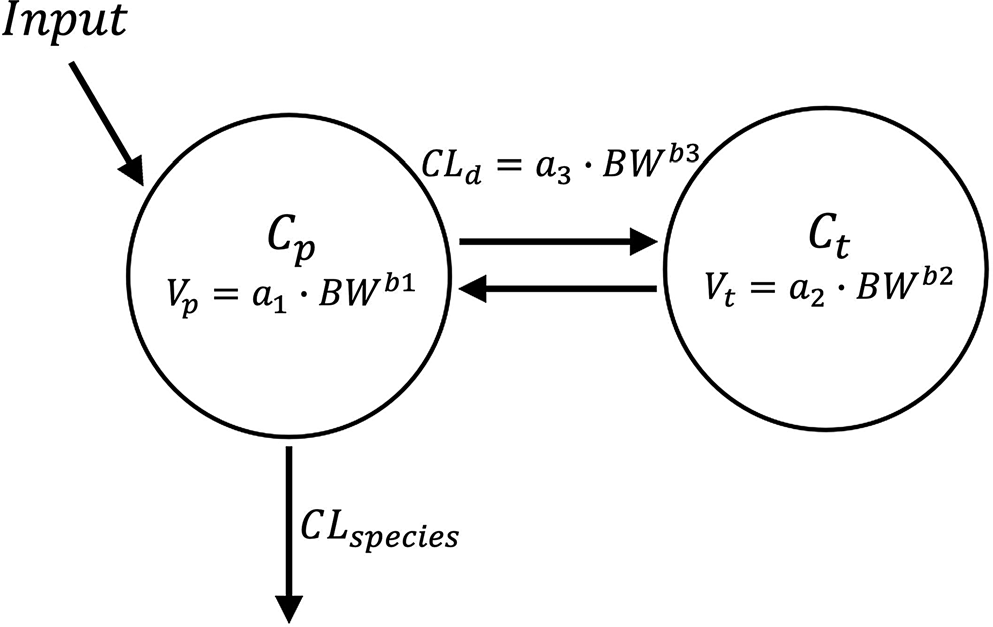
Two-compartment model (2CM) structure with allometric parameter equations. Symbols are defined in Table 2

**Table 2:** PK parameters estimated by joint fitting of 8 species data to the 2CM with the allometric relationships for kinetic parameters ($\:{V}_{p}$, $\:{V}_{t}$, and $\:C{L}_{d}$). For humans, the $\:{k}_{a}$ was estimated, with the $\:F$ value fixed (see text). The model fittings are shown in Fig. 5

Parameter	Definition (units)	Estimates (CV%)
$\:{a}_{1}$	Intercept for the volume of central compartment, $\:{V}_{p}$ (L)	0.339 (12.68)
$\:{b}_{1}$	Exponent for the volume of central compartment, $\:{V}_{p}$ (unitless)	1.309 (3.33)
$\:{a}_{2}$	Intercept for the volume of peripheral compartment, $\:{V}_{t}$ (L)	0.937 (21.13)
$\:{b}_{2}$	Exponent for the volume of peripheral compartment, $\:{V}_{t}$ (unitless)	0.885 (8.33)
$\:{a}_{3}$	Intercept for distributional clearnace, $\:C{L}_{d}$ (L/h)	0.352 (28.20)
$\:{b}_{3}$	Exponent for distributional clearance, $\:C{L}_{d}$ (unitless)	0.920 (10.89)
$\:{k}_{a}$	Absorption rate constant for human oral dose (1/h)	1.702 (12.10)
$\:F$	Bioavailability for human oral dose (unitless)	0.66 (Fixed)

Note: The model-fitted species-specific $\:CL$ values are listed in Table 1

**Fig. 3:**
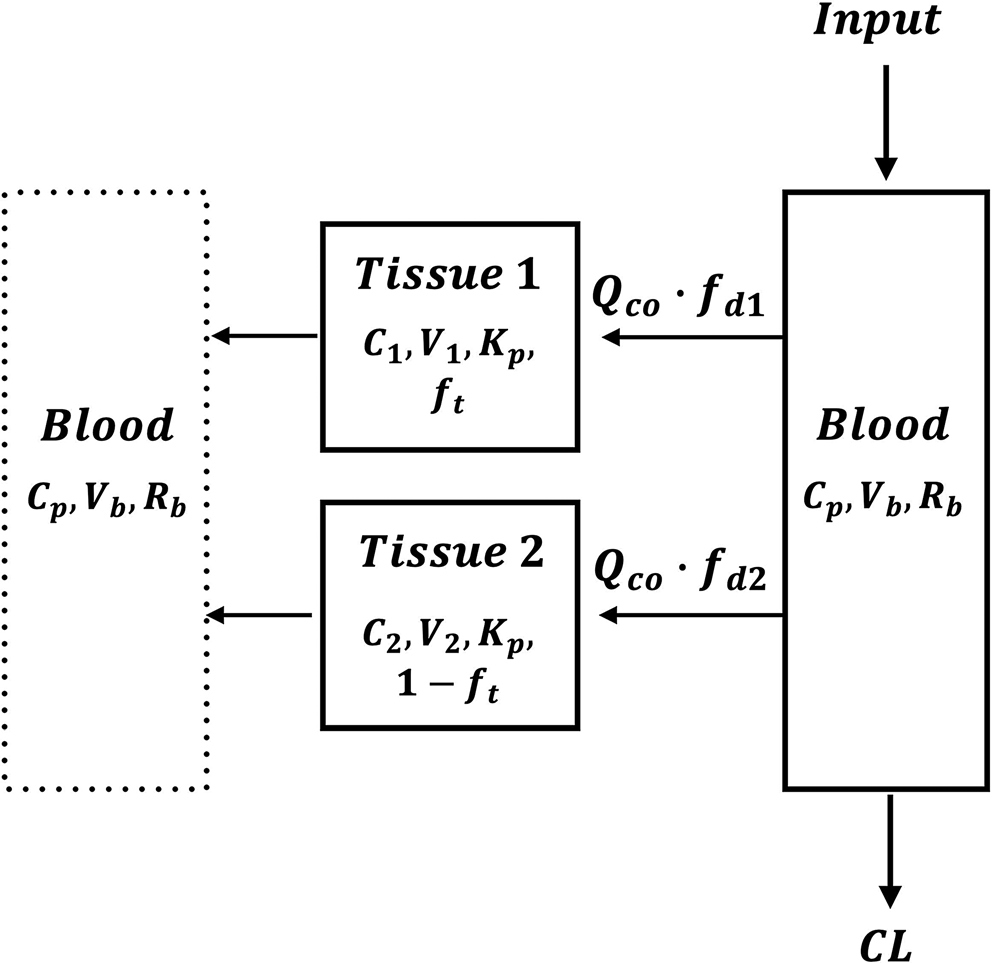
Minimal physiologically based pharmacokinetic model (mPBPK) structure with two tissue compartments for an IV bolus input into the blood compartment. A single $\:{K}_{p}\:$parameter $\:\left({K}_{p1}={K}_{p2}\right)$ was used in the joint model fitting, with the cardiac output ($\:{Q}_{co}$) calculated based on the allometric relationship (Eq. (8)), as listed in Table S1C for each species. Parameter definitions are provided in the text and Table 3

**Table 3:** PK parameters for the mPBPK model (perfusion-limited distribution;$\:{f}_{d,total}=1$) jointly fitted to PK data across 8 species. Species-specific$\:{K}_{p}$values were used for pigs and chickens. The model fittings are shown in Fig. 6

Parameter	Definition	Estimates (CV%)
$\:{f}_{t}$	Fraction of total tissue volume for tissue 1	0.497 (2.71)
$\:{f}_{d,total}$	Total sum of fraction distribution parameters ($\:{f}_{d1}+{f}_{d2}$)	1.0
$\:{f}_{d1}$	Fraction distribution of$\:{Q}_{co}$for tissue 1	0.038 (5.75)
$\:{f}_{d2}$	Fraction distribution of$\:{Q}_{co}$for tissue 2	0.962^b^
$\:{K}_{p}$	Tissue-to-plasma partition coefficient	1.41 (2.40)
$\:{K}_{p,pig}$	Specific tissue-to-plasma partition coefficient of pig	5.62 (7.71)
$\:{K}_{p,chicken}$	Specific tissue-to-plasma partition coefficient of chicken	3.38 (4.47)

^a^Species-specific$\:CL$values from joint mPBPK model fittings are listed in Table 1

^b^The value of$\:{f}_{d2}$was calculated as$\:{f}_{d,\:total}-{f}_{d1}$

**Fig. 4:**
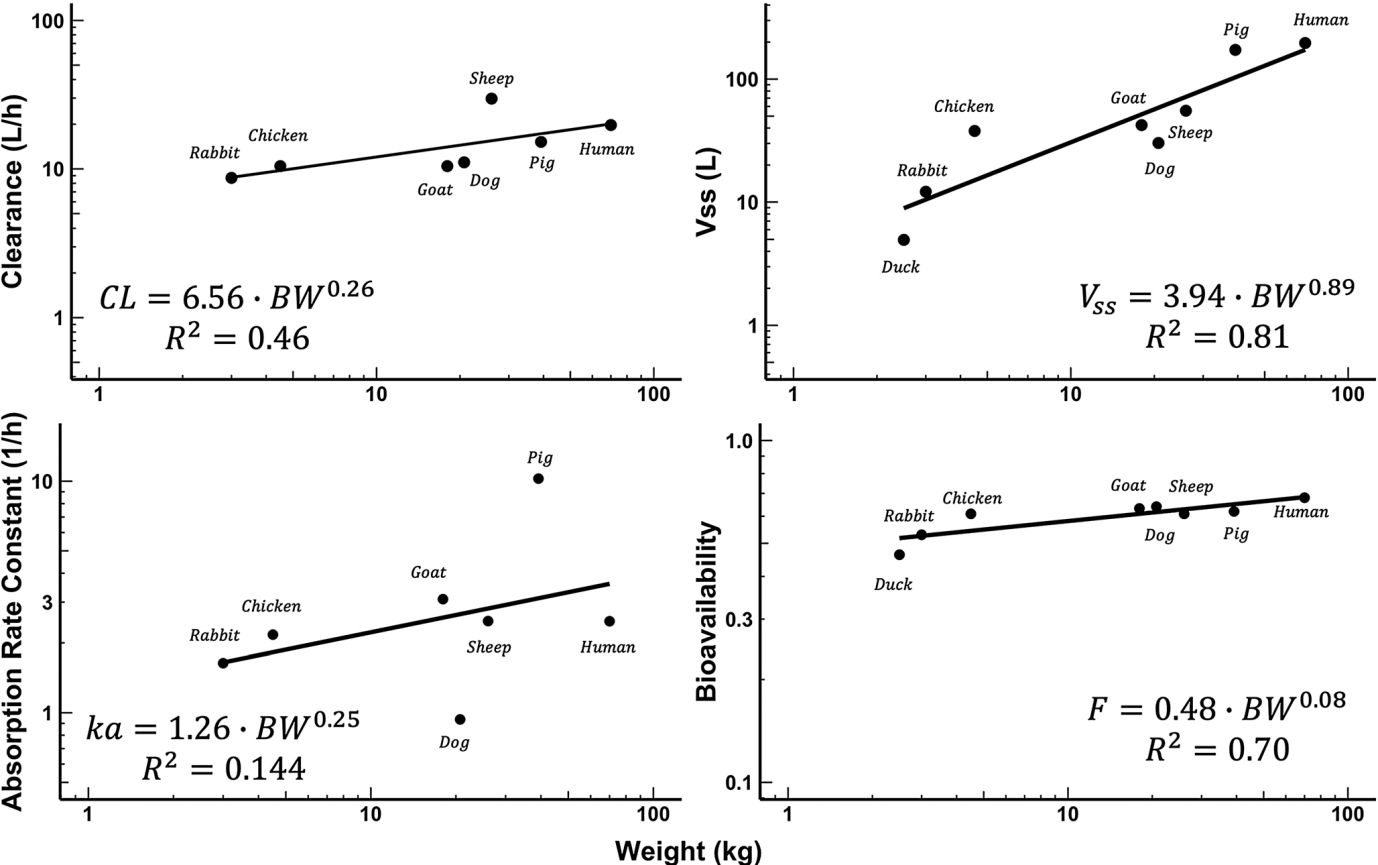
Allometric relationships for indicated PK parameters versus body weights. The PK parameters were calculated by NCA as primarily obtained from the literature

**Fig. 5:**
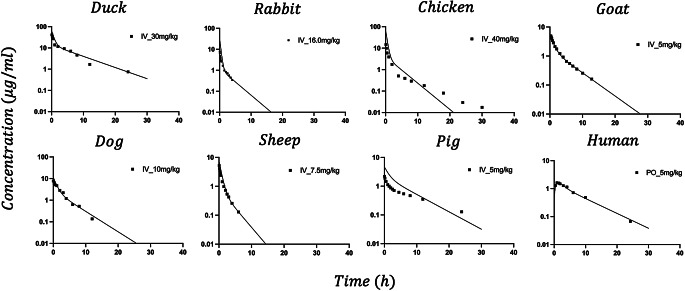
Model fittings for the 2CM jointly fitted to IV PK data for 8 species. Parameter estimates are listed in Table 2

**Fig. 6:**
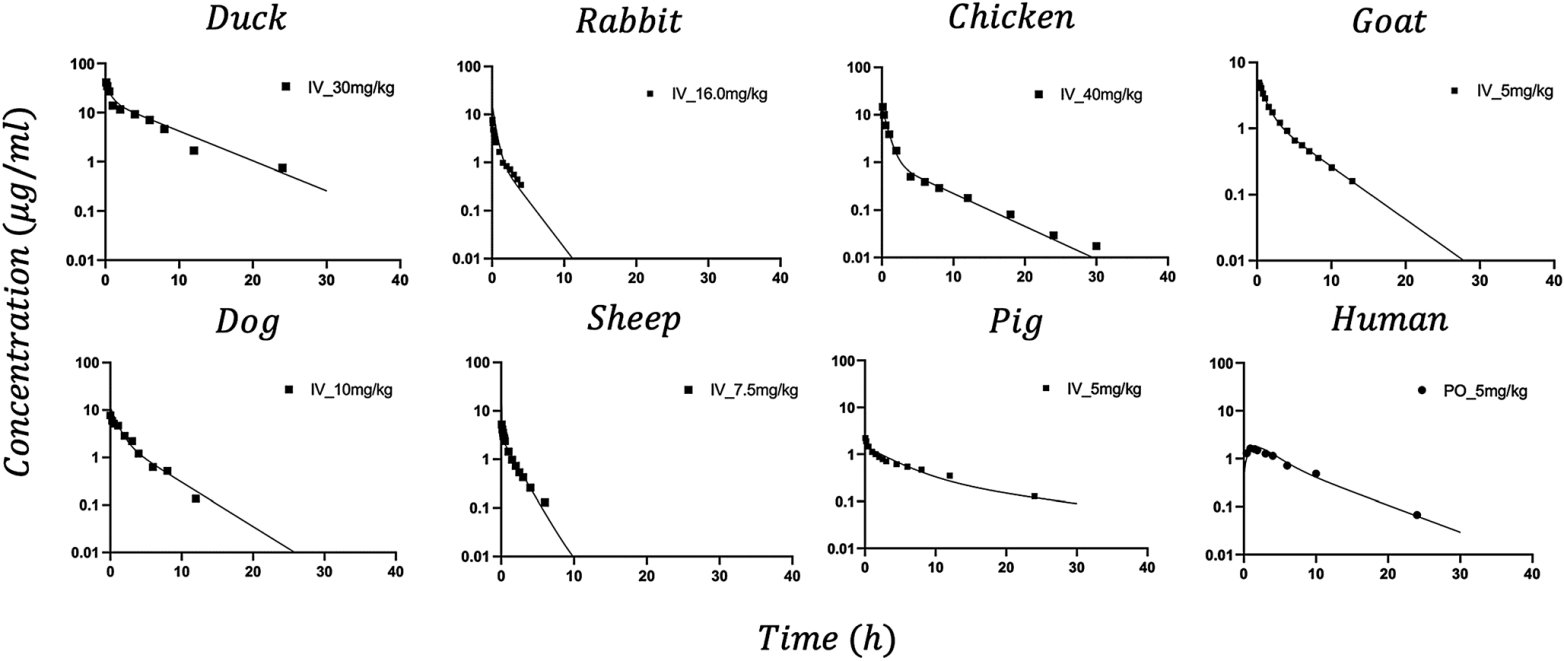
Model for the mPBPK model jointly fitted to the IV PK data for 8 species. Common $\:{K}_{p}$ and species-specific $\:{K}_{p,pig}$ and $\:{K}_{p,chicken}$ and other disposition kinetic parameters ($\:{f}_{t}$, $\:{f}_{d1}$, and $\:{f}_{d2}$) are listed in Table 3

### Data collection

PubMed (https://pubmed.ncbi.nlm.nih.gov/) and Google Scholar (https://scholar.google.com/) were used to identify relevant publications. The search strategy involved the following keywords and their synonyms combined with the ‘OR’ operator: (i) Levamisole, (ii) pharmacokinetics, (iii) distribution, (iv) metabolism, and (v) species. These keywords were then interconnected using the ‘AND’ operator. Additionally, reference tracing was conducted to ensure thorough coverage. The publication timeframe spanned from 1930 to 2025, capturing both historical and recent studies, as LVM has been a well-established drug for decades.

### Calculation of basic PK parameters

The PK data for LVM were identified for 18 species, with 8 species suitable for further allometric scaling. When PK parameters were not directly reported in the original articles, published concentration-time data were digitized using WebPlot Digitizer (https://automeris.io/WebPlotDigitizer). Key PK parameters, including clearance ($\:CL$), extravascular clearance [$\:CL/F$; oral (PO)/intramuscular (IM), subcutaneous (SC)], steady-state volume of distribution ($\:{V}_{ss}$), area under the curve from time 0 to infinity ($\:{AUC}_{0}^{\infty\:})$, terminal-phase half-life ($\:{T}_{1/2}$), and bioavailability ($\:F$), were primarily calculated using non-compartmental analysis (NCA) in Phoenix WinNonlin version 8.4 (Certara USA, Inc., Princeton, NJ). The classic allometric equation ($\:Y=a\cdot\:B{W}^{b}$) (Adolph 1949) relating PK parameters ($\:Y$) to body weights ($\:BW$) was initially applied to available PK parameters.

The absorption rate constant$\:\:\left({k}_{a}\right)$ and bioavailability ($\:F$) were estimated either by moment analysis or model fitting. In moment analysis, the area under the first moment curve from time 0 to infinity ($\:AUM{C}_{0}^{\infty\:}$) was divided by the area under the curve ($\:{AUC}_{0}^{\infty\:}$) to obtain the mean residence times of LVM in the body after its extravascular and intravenous (IV) administration (i.e., $\:MR{T}_{EV}$ and $\:MR{T}_{IV}$) (Jackson and Chen 1987). The mean absorption time ($\:MAT$) was obtained as their difference (Eq. (1), and the $\:{k}_{a}$ was calculated as 1/$\:MAT$.

1 $$\:MAT=MR{T}_{EV}-MR{T}_{IV}$$

The hepatic availability ($\:F$*) of LVM was estimated by assuming that LVM is mainly metabolized in liver (Gibaldi et al. 1971):

2 $$\:{F}^{*}=1-C{L}_{H}/({Q}_{H}\cdot\:{R}_{b})$$

where $\:C{L}_{H}$ is the hepatic clearance (with respect to the plasma concentration), which was calculated as $\:Dose/AUC$ or estimated by model fitting, $\:{Q}_{H}$ denotes the hepatic blood flow for each species, and $\:{R}_{b}$ is the blood-to-plasma partition coefficient, which was assumed to be 1. Parameters were assessed by the basic allometric relationship ($\:Y=a\cdot\:B{W}^{b}$) using R studio (https://www.R-project.org/).

### Model-based assessment of LVM PK

The plasma concentration-time data of LVM were collected and a series of different modeling strategies were tried depending on the data types: assessment of (i) only IV data for each individual species and (ii) IV and oral data combined for each individual species, and (iii) joint assessment of the IV data for all available species based on a consideration of allometric relationships. A typical two-compartment model (2CM) (Fig. 2) or a typical minimal PBPK (mPBPK) model (Fig. 3) was considered for each data array (i.e., 6 combinations). For the joint PK assessment (iii), the allometric relationship was applied for the 2CM distribution parameters and for the cardiac output in the mPBPK.

### Two-compartment model (2CM)

The LVM concentration-time data were fitted to the 2CM. The differential equations and initial conditions after a bolus IV dose are:

3 $$\:{V}_{p}\cdot\:\frac{d{C}_{p}}{dt}=C{L}_{d}\cdot\:\left({C}_{t}-{C}_{p}\right)-CL\cdot\:{C}_{p}\:\,\,\,\,\,\,\,\,\,\,\,\,\,\,\,\,{C}_{p}\left(0\right)=Dose/{V}_{p}$$

4 $$\:{V}_{t}\frac{d{C}_{t}}{dt}=C{L}_{d}\cdot\:\left({C}_{p}-{C}_{t}\right)\,\,\,\,\,\,\,\,\,\,\,\,\,{C}_{\mathrm{t}}\left(0\right)=\:0$$

where $\:{C}_{p}$ and $\:{C}_{t}$ are the LVM concentrations in the central ($\:{V}_{p}$) and peripheral volumes ($\:{V}_{t}$), $\:C{L}_{d}$ is the distributional clearance between these compartments, and $\:CL$ denotes the elimination clearance from plasma. The steady-state volume of distribution is $\:{V}_{ss}={V}_{p}+{V}_{t}$.

For joint assessment, we assumed that the distribution parameters, i.e., $\:{V}_{p}$, $\:{V}_{t}$, and $\:C{L}_{d}$, have an allometric relationship ($\:a\cdot\:B{W}^{b}$), and the species-specific $\:CL$ values were considered. The intercepts and exponents used are $\:{a}_{1}$ and $\:{b}_{1}$ for $\:{V}_{p}$, $\:{a}_{2}$ and $\:{b}_{2}$ for $\:{V}_{t}$, and $\:{a}_{3}$ and $\:{b}_{3}$ for $\:C{L}_{d}$.

### Minimal PBPK (mPBPK) model

The across-species PK data for LVM were assessed using a typical mPBPK model (Fig. 3). The differential equations for the IV bolus dose are:

$$\begin{aligned}&{V}_{b}{R}_{b}\cdot\:\frac{d{C}_{p}}{dt}\cr&={Q}_{co}\cdot\:{f}_{d1}\cdot\:{R}_{b}\cdot\:\left(\frac{{C}_{1}}{{K}_{p}}-{C}_{p}\right)\cr&+{Q}_{co}\cdot\:{f}_{d2}\cdot\:{R}_{b}\cdot\:\left(\frac{{C}_{2}}{{K}_{p}}-{C}_{p}\right)-CL\cdot\:{\mathrm{C}}_{p}\end{aligned}$$

5 $$\:\:{C}_{p}\left(0\right)=Dose/\left({V}_{b}{R}_{b}\right)$$

6 $$\:{V}_{1}\frac{d{C}_{1}}{dt}={Q}_{CO}\cdot\:{f}_{d1}\cdot\:{R}_{b}\cdot\:\left({C}_{p}-\frac{{C}_{1}}{{K}_{p}}\right)\,\,\,\,\,\,\,\,\,\,\,{C}_{1}\left(0\right)=0$$

7 $$\:{V}_{2}\frac{d{C}_{2}}{dt}={Q}_{CO}\cdot\:{f}_{d2}\cdot\:{R}_{b}\cdot\:\left({C}_{p}-\frac{{C}_{2}}{{K}_{p}}\right)\,\,\,\,\,\,\,\,{\:C}_{2}\left(0\right)=0$$

where $\:{C}_{p}$ is the plasma concentration of LVM, $\:{C}_{1}$ and $\:{C}_{2}$ are the LVM concentrations in tissues 1 and 2, $\:CL$ is the species-specific systemic clearance, and $\:{K}_{p}$ is the tissue-to-plasma partition coefficient. The blood volumes ($\:{V}_{b}$) for duck, rabbit, chicken, goat, dog, sheep, pig, and humans were from literature sources (Table S1A). The cardiac output ($\:{Q}_{co}$) (Table S1B) was calculated (Brown et al. 1997) based on:

8 $$\:{Q}_{CO}\:\left(L/h\right)=14.1\cdot\:BW{\left(kg\right)}^{0.75}$$

where body weights ($\:BW$) were from the original publications or assumed as typical (Table S1C).

Assuming the density of the tissues is unity, the anatomical tissue volumes 1 ($\:{V}_{1}$) and 2 ($\:{V}_{2}$) were calculated by multiplying the total tissue weight ($\:=BW-{V}_{b}$) by the volume fraction of the tissues 1 ($\:{f}_{t}$) and 2 ($\:1-{f}_{t}$):

9 $$\:{V}_{1}=\left(BW-{V}_{b}\right)\cdot{f}_{t}$$

10 $$\:{V}_{2}=\left(BW-{V}_{b}\right)\cdot\:\left(1-{f}_{t}\right)$$

The $\:{f}_{d1}$ and $\:{f}_{d2}$ in Eqs. (5–7) denote the fractional distribution parameters for the tissues 1 and 2. According to the Biopharmaceutical Drug Disposition Classification System (BDDCS), LVM has high permeability in light of its extensive metabolism (Kambayashi et al. 2023). However, the World Health Organization (WHO) assigns levamisole to BCS Class III. Considering this ambiguity, the permeability factor for LVM was fitted based on:

11 $$\:{f}_{d,total}={f}_{d1}+{f}_{d2}\le\:1$$

For both individual- and joint-species modeling, the primary estimated parameters were $\:CL$, $\:{K}_{p}$, $\:{f}_{t}$, $\:{f}_{d,total}$, and $\:{f}_{d1}$, with $\:{f}_{d2}$ being a secondary parameter. For the joint assessment, a species-specific $\:{K}_{p}$ was applied for chickens and pigs, reflecting their unique tissue distribution or composition (Björkman 2003; Poulin and Theil 2000).

## Absorption kinetics

The LVM kinetics in the absorption compartment was described as:

12 $$\:\:\frac{d{A}_{a}}{dt}=\:-{k}_{a}\cdot\:{A}_{a}\:\,\,\,\,\,\,\,\,\,{A}_{a}\left(0\right)=\:Dose$$

where $\:{A}_{a}$ is the LVM amount in the absorption compartment and $\:{k}_{a}$ is the absorption rate constant. The absorption rate of LVM (i.e., $\:F\cdot\:{k}_{a}\cdot\:{A}_{a}$) was added to the right-hand side of Eq. (3) for the 2CM or Eq. (5) for the mPBPK, with their initial conditions set to 0. Since IV data were not available for humans, the joint fitting of the 8 species data was done by estimating the human $\:{k}_{a}$ and fixing the human $\:F$ value as 0.66 based on the literature.

### Model fitting

Model fittings were conducted using the maximum likelihood method in ADAPT (D’Argenio et al. 2009), with the variance of $\:{V}_{i}={\left({\sigma\:}_{1}+{\sigma\:}_{2}\cdot\:{Y}_{i}\right)}^{2}$ where $\:{V}_{i}$ is the variance of the $\:i$th data point, $\:{Y}_{i}$ is the $\:i$th model prediction results, and $\:{\sigma\:}_{1}$ and $\:{\sigma\:}_{2}$ are the variance model parameters. The goodness-of-fit was assessed based on the visual inspection, Akaike Information Criterion (AIC), and Coefficient of Variance (CV%). The ADAPT 5 codes for the 2CM and mPBPK models are consistent with those in Song and Jusko (2021). Phoenix WinNonlin was used for NCA. Figures were generated using GraphPad Prism 10.2.

## Results

### Data collection and basic allometric assessment of PK parameters

The references and PK data for 18 species are listed in Table S2 (IV data) and Table S3 (oral data). Human PK data are summarized in Table S4. The data from 8 species were selected for model fittings based on the following criteria: (i) Availability of IV data (except for humans) and (ii) exclusion of cold-blooded species, as their basal metabolic rate (BMR) is known to be significantly low (McNab 2019). When multiple PK profiles were available for a given species, profiles with longer collection times or more comprehensive data were prioritized. For species with a range of doses (e.g., rabbits and sheep), the middle values were selected.

Figure 4 shows log-log plots of published PK parameters in relation to body weight along with their corresponding allometric regression parameters. The reported $\:CL$ values of LVM exhibited a simple allometric relationship with body weight ($\:b$ = 0.26) across 7 species, excluding the duck. The reported $\:{V}_{ss}$ values have an excellent allometric relationship ($\:b$ = 0.89) across 8 species. In contrast, the absorption rate constants ($\:{k}_{a}$) showed a poor allometric relationship ($\:b$ = 0.25) with a low R^2^ value. The bioavailability ($\:F$) displayed a weak relationship ($\:b$ = 0.08), where the near-zero $\:b$ value indicates a constant $\:F$ value of 0.463 to 0.666 ($\:a$ = 0.48).

### Two-compartment model fitting

The digitized PK profiles of LVM were initially assessed by applying the 2CM to each of the 8 species data, using the IV data only ($\:{V}_{p}$, $\:{V}_{t}$, $\:C{L}_{d}$, and $\:CL$; Table S5) and for the IV and oral data combined ($\:{V}_{p}$, $\:{V}_{t}$, $\:C{L}_{d}$, $\:CL$, $\:{k}_{a}$, and $\:F$; Table S6). The parameter estimates aligned well with those in the original publications. As shown in Figures S1 (IV only, except for human) and S2 (IV and oral combined), most species showed reasonable agreement between the observed data and the model fitting results. Table 1 summarizes the $\:CL$ values reported in the literature and estimated by different modeling approaches.

Figure 5 shows the PK data for 8 species jointly fitted to the allometric 2CM model. The estimates of $\:{a}_{i}$ and $\:{b}_{i}$, and the definition of symbols are listed in Table 2. The small CV% shows reasonable model performance. The Akaike Information Criterion (AIC) of the model is 598,512. The exponent $\:b$ ranged from 0.885 to 1.30, indicating a relationship that was close to, but not strictly proportional to $\:BW$. The $\:{V}_{t}$ primarily determines $\:{V}_{ss}$, and their estimated $\:b$ values appear nearly identical (i.e., 0.885 for $\:{V}_{t}$ versus 0.89 for $\:{V}_{ss}$; Fig. 4). The PK fittings for the chicken and pig have significant discrepancies.

### Minimal PBPK model fitting

The results of fitting of the mPBPK model to each species PK data are shown in Figures S3 (IV data only) and S4 (IV and oral data combined). The modeling of the IV data (Figure S3) well captured the observed data, but some underestimations occur in the fitting of IV and oral data in pig (Figure S4). The parameter estimates are provided in Tables S7 (IV data only, except for human) and S8 (IV and oral data combined). While low CV% values support reasonable model performance for most species, an apparently mono-phasic decline after IV dosing in dogs (Figure S3) and the absence of the early-phase data after oral dosing in goats (Figure S4) yielded some uncertainties in the parameter estimates in dogs (e.g., $\:{f}_{d1}$ and $\:{f}_{t}$) and in goats (e.g., $\:F$).

The fittings of the IV data with the mPBPK model for all species at one time with the optimized model is shown in Fig. 6 with the model parameters listed in Table 3. The PK profiles were reasonably captured showing the general poly-exponential decline. The assumption of low tissue permeability yielded unsuitable mPBPK fittings, which argued for use of the perfusion-limited model ($\:{f}_{d1}+{f}_{d2}=1$). In addition, incorporating one globally-applicable $\:{K}_{p}$ value (1.41) along with species-specific $\:{K}_{p}$ values for chicken (3.38) and pig (5.61) provided better curve fitting with lower CV% values, compared to using a single unified $\:{K}_{p}$ across all species, which yielded an AIC of 598,377. Although the minipig has often been considered a reasonable model for human absorption and clearance, its distribution characteristics have not been explored (Tang and Mayersohn 2018) and making its typical distribution behavior unclear.

A comparison of species-specific $\:CL$ values with previously estimated model results, as shown in Table 1 and Figure S5, reveals notable diversity. However, experimental values for other distribution parameters are lacking, limiting direct comparisons with the present fitting results.

## Discussion

LVM is widely used in both human and veterinary medicine owing to its anti-parasitic, immunomodulatory, and anti-inflammatory effects. Despite its extensive clinical and agricultural applications, a systematic interspecies assessment of its PK has not been conducted. By analyzing PK data from over 40 publications across 18 species, this study employs allometric scaling and a series of modeling approaches to provide new and generalized insights into the interspecies PK characteristics of LVM. Of note, we did not include rodents in the current PK analysis due to the absence of suitable IV PK profiles for LVM in rats and mice in the literature. The paucity of rodent data is likely due to limited use of LVM in rodent veterinary studies.

### Allometric scaling

Traditional allometric scaling was first applied to assess calculated NCA parameters obtained from literature. The reported $\:CL$ values of LVM across 7 species showed a small power coefficient ($\:b$ = 0.26) with $\:BW$, as depicted in Fig. 4. Notably, this appears to be the lowest $\:b$ value among any compound reported (Tang and Mayersohn 2005), emphasizing the unique PK of LVM. However, the relatively low R^2^ value (Fig. 4) precluded its use for allometric $\:CL$ estimation in joint fittings with the 2CM and mPBPK models. The low renal $\:CL$ and high metabolic $\:CL$ of LVM may have contributed to a weak R^2^ value observed for $\:CL$ (Fig. 4), as suggested by (Tang and Mayersohn 2006). These findings highlight the limitations of traditional allometric scaling in capturing interspecies variability in LVM PK, underscoring the need for improved modeling approaches.

### Model comparisons and performance

Our joint modeling techniques were used to analyze LVM PK profiles across 2 bird and 6 mammalian species. Although various fish species with unique elimination mechanisms such as gill diffusion have also been studied for LVM PK (Tables S2 and S3), cold-blooded species were excluded from the current analysis, partly because most allometric scaling studies relevant to human translation focus on mammalian species. A single-dose representative PK profile was selected per species to ensure equal weighting in model fittings, while all available data were used for joint fittings of IV and oral profiles.

The 2CM and mPBPK models allowed a joint evaluation of full PK profiles across species by incorporating allometric scaling and physiological parameters. The 2CM applied allometric scaling to distribution parameters (Fig. 2), whereas the mPBPK model utilized species-specific physiological variables such as blood volume, cardiac output, and body weight (Fig. 3). The main interspecies difference was observed in $\:{K}_{p}$ values: Chickens and pigs displayed markedly higher $\:{K}_{p}$ values than other species, suggesting greater tissue partitioning in these species. While the mPBPK model adequately captured LVM PK profiles across most species (Fig. 6), the poor CV% values observed in dogs likely resulted from profiles displaying only a single exponential phase.

In both whole-body PBPK and mPBPK models, a high initial blood concentration is typically expected after IV dosing because of the small blood volume. Therefore, these model-predicted concentrations produce a larger $\:AUC$ and a slightly lower $\:CL$ than does the 2CM. Species-specific $\:CL$ estimates were consistent between models for most species, except for rabbits and chickens, likely due to the lack of early time-concentration data (Figure S5).

Our joint mPBPK model was applied to 8 species, comparable to previous studies with dexamethasone (11 species) and metformin (9 species) (Jeong and Jusko 2021; Song and Jusko 2021). The current analysis for LVM provided reasonable fittings and low CV% for most 2CM and mPBPK model parameters. In addition, the estimated $\:CL$ values were largely consistent with those reported in the literature (Table 1 and Figure S5). The minimal PBPK model yielded a substantially lower AIC (598,377) than the allometric 2CM (598,512).This supports the use of mPBPK as the more parsimonious yet better-fitting model for describing interspecies LVM PK.

### Absorption kinetics

Given its high aqueous solubility (210 mg/mL), LVM is unlikely to exhibit solubility-limited intestinal absorption at typical oral doses (e.g., 50 to 150 mg; Table S4) administered with 250 mL of water. However, little evidence on the intestinal permeability of LVM in the literature has led to a conservative BCS classification (e.g., BCS Class III) for this drug (Kambayashi et al. 2023). One study anecdotally reported that LVM exhibited higher permeability than the high-permeability marker metoprolol in both in vitro cell monolayer and in situ chicken intestinal perfusion studies (Xiangxiu et al. 2020), suggesting high intestinal permeability. Assuming no species difference in the effective intestinal permeability of LVM ($\:{P}_{eff}$ = 0.93 × 10^− 4^ cm/s), and applying a 7-compartment intestinal absorption model (Lawrence and Amidon 1999), the fraction absorbed ($\:{F}_{a}$) from the human intestinal lumen was estimated using the equation $\:{F}_{a}=1-{(1+0.54{P}_{eff})}^{-7}$, yielding a value of 0.688. When multiplied by the estimated hepatic availability ($\:{F}^{*}$ = 0.8; Table S1B), the resulting oral bioavailability (~55%) was consistent with reported values ranging from 62.5 to 68% (Table S9). LVM is rapidly absorbed in most species via oral, IM, and SC routes, with the observed $\:{T}_{max}$ values ranging from 0.25 to 1.94 h (Table S3). Notably, bulls (IM), dogs (PO/SC), calves (PO/SC), and pigs (SC) showed peak blood concentrations within 0.25 to 0.5 h following oral doses, which further supports the rapid absorption of LVM. However, oral bioavailability ($\:F$) varied moderately across species (Table S9 and Fig. 4), ranging from 43.2% in beluga (huso-huso) to 88% in chickens, while humans have reported values between 62.5 and 68%. This incomplete bioavailability can be attributed, at least in part, to first-pass hepatic elimination, as non-renal $\:CL$ accounts for 95% of total LVM elimination in humans. Interestingly, LVM exhibits flip-flop kinetics in rabbits: the PK profile shows a slower up-curve with a later $\:{T}_{max}$ compared to other species, and the terminal-phase half-life after PO dosing exceeds that seen after IV administration (Figures S2 and S4). Rabbits also demonstrate low hepatic extraction (Villanueva et al. 2003), suggesting a significant contribution of non-hepatic clearance pathways in this species.

### Distribution kinetics

LVM was found to follow a perfusion-limited distribution model. The fractional distribution parameter $\:{f}_{d}$ represents the proportion of drug transported into tissues during a single pass of blood. When tissue permeability is sufficiently higher than local blood flow, the total distributional clearance approaches the cardiac output, as reflected by the condition that the sum of $\:{f}_{d}$ values equals 1 in Eq. (11) (Jeong et al. 2017). Notably, a perfusion-limited model has also been reasonably applied to metoprolol in rats (Kir et al. 2025), which has been reported to exhibit lower permeability coefficients than LVM. This finding supports our use of a perfusion-limited model to describe the distribution kinetics of LVM. Consistent with this, since $\:{f}_{d}$ serves as the upper limit for the organ extraction ratio (Jeong and Jusko 2022), the high $\:{f}_{d}$ values of LVM are in good agreement with its observed extensive metabolism (see below).

The reported steady-state volumes of distribution ($\:{V}_{ss}$) surprisingly ranged widely from 0.81 to 8.36 L/kg, with most values falling between 1 and 4 L/kg (Table S2). Tritium-associated radioactivity of LVM was higher than its non-labeled plasma concentration in rats, pigs, ewes, and goats after IM doses, indicating extensive metabolism. Rapid and extensive tissue distribution of LVM radioactivity was found in rats, dogs, and monkeys, with the liver and kidneys showing the highest radioactivity in cattle and pigs (Koyma et al. 1983). LVM and its metabolites can be excreted into eggs and milk of laying hens and lactating cows (Paulson and Feil 1996). Although plasma protein binding in chickens (19.4%) and pigs (24.7%) is comparable to that observed in other species (22.4–25.9%) (Sahagún et al. 1997), their $\:{V}_{ss}$ values (from NCA; 8.07 and 4.73 L/kg; Table S2) are unusually high. Therefore, species-specific $\:{K}_{p}$ values were needed, since factors beyond plasma protein binding (e.g., tissue partitioning) likely account for these higher volumes. In dogs, plasma protein binding is extensive (80–95%; (Plante et al. 1981), likely contributing to a relatively low $\:{V}_{ss}$ of 1.69 L/kg (Table S2).

In mPBPK modeling, tissues are grouped into “tissue 1” and “tissue 2” compartments based on differences in distribution characteristics. (Jeong et al. 2022a, b) proposed a systematic method for assigning tissues to each peripheral mPBPK compartment by calculating their mean transit times ($\:MTT$), where $\:MTT$ is defined as the time for drug molecules to pass through a tissue once. This approach requires knowledge of the rate ($\:{f}_{d}$) and extent ($\:{K}_{p}$) of drug distribution in each tissue. Because little LVM tissue distributiondata are available for any of the species examined, we could not estimate tissue-specific $\:{f}_{d}$ or $\:{K}_{p}$ values and therefore were unable to apply this tissue-lumping framework.

### Metabolism and clearance

LVM undergoes extensive metabolism across species, primarily via oxidation and hydrolysis, with partial excretion in urine. In humans, *p*-hydroxylation is a key metabolic pathway. In cows, approximately 80% of orally administered LVM (measured as ^14^C-activity) is excreted in urine, with no detectable unchanged parent drug (Paulson and Feil 1996). In goats, around 55% of the administered dose is excreted in urine as LVM and its metabolites, while about 30% is recovered in feces. In rats, 32–45% of plasma radioactivity corresponds to unchanged LVM, and 46% of the radioactivity is excreted in urine within 24 h, indicating only partial metabolism (Plante et al. 1981). In pigs, extensive metabolism results in 80–85% of the dose being excreted in urine, with only 5–10% found in feces. In dogs, renal elimination involves tubular secretion mediated by the organic base transporter (Plante et al. 1981).

A psychoactive metabolite, aminorex, is of particular concern when LVM is used as a cocaine adulterant due to its structural and functional similarities to amphetamines, which inhibit neurotransmitter transporters and exert psychostimulant effects (Hofmaier et al. 2014). In horses, aminorex has been detected in urine for up to 16 days following LVM administration, highlighting its relevance in doping control in racehorses (Philip et al. 2023). However, aminorex concentrations in plasma could not be found in the literature.

The reported $\:CL$ values for LVM vary widely, ranging from 0.14 to 3.21 L/h/kg across reptiles, fish, birds, and mammals (Table S10). Reptiles show $\:CL$ values of 0.14 (red-eared slider turtle) and 0.22 L/h/kg (Caspian turtle), which are similar to the value observed in fish (Huso huso, 0.31 L/h/kg). Environmental factors such as temperature and salinity, may contribute to these similarities. In birds, ducks exhibit a relatively low $\:CL$ of 0.27 L/h/kg, whereas prelay chickens have a much higher $\:CL$ of 2.46 L/h/kg. These differences might be attributed to interspecies physiological variations in renal morphology and liver enzyme activity between waterfowl and galliform birds (Sartini et al. 2021) In humans, oral $\:CL$ values range from 0.25 to 0.51 L/h/kg, based on the data from both healthy individuals and cancer patients (Table S4).

After completion of our PK modeling assessment, an additional recent study of levamisole PK was found in dogs reflective of the continued high interest in therapeutic use of this drug. AUCs ranging from 2999.5 to 30,387.5 ng·h/mL at doses of 5–30 mg/kg were found (Zhang et al. 2024). In our meta-analysis, the estimated $\:{AUC}_{0}^{\infty\:}$ following a 10 mg/kg dose of levamisole in dogs was 12,180 ng·h/mL.

### Study limitations

This study has limitations related to the completeness of available data and the variability in analytical methods used across reports. The blood-to-plasma partition coefficients ($\:{R}_{b}$) and protein binding values were unavailable for many species, limiting the ability to further refine species-specific distribution parameters. Some PK parameters were estimated using NCA based on digitized concentration-time graphs that assumes that the observed profiles were typical. All PK fittings were performed under the assumption of linear absorption and disposition, although this was supported by dose-ranging data in some species. While our study was not intended for predictive purposes, the inclusion of PK data from 18 species, many of which provided both IV and oral profiles, offered a unique and valuable opportunity to characterize the interspecies PK properties of this important therapeutic agent.

## Conclusion

LVM is widely recognized for its significant antiparasitic activity and has been used in a broad range of species, including mammals, fish, and reptiles. We offered a flow chart for systematic performance of mPBPK studies and summarized all available PK data and utilized datasets from 8 species to apply both 2CM and mPBPK models with allometric scaling. The PK in cold-blooded species was comparable to that of mammals, but with consistently lower $\:CL$ values. The $\:CL$ of LVM showed a uniquely low allometric exponent ($\:b$) across species, whereas tissue distribution parameters such as $\:{V}_{ss}$ were highly variable but generally followed $\:BW$ proportionality. Joint model fittings using the 2CM and mPBPK approaches yielded reasonable performance in most species, though notable exceptions were observed. These differing PK behaviors included the low $\:F$ in goats and the unusually high $\:{K}_{p}\:$values in chickens and pigs. Overall, LVM displayed some distinctive yet relatively consistent PK characteristics across the studied species, highlighting its utility for interspecies PK modeling.

## Supplementary Information

Below is the link to the electronic supplementary material.

Supplementary Material 1

## Data Availability

All data generated or analyzed during this study are included in this published article [and its supplementary information files].
