# Supplementary material for: Meta-analysis of levamisole absorption and disposition across diverse species using a minimal physiologically-based pharmacokinetic model
Source: J Pharm Investig. 2025 Sep 11;56(1):171–83. doi: 10.1007/s40005-025-00770-6 (PMC12769651; doi:10.1007/s40005-025-00770-6)
Supplement: Supplementary file 1 — Supplementary Material 1 [file 40005_2025_770_MOESM1_ESM.docx]

**Supplemental Materials**

**Meta-analysis of levamisole absorption and disposition across diverse species using a minimal physiologically-based pharmacokinetic model**

ChunFu Cheng, Yoo-Seong Jeong*, and William J Jusko*

Department of Pharmaceutical Sciences, School of Pharmacy and Pharmaceutical Sciences, State University of New York at Buffalo, Buffalo, New York

***Co-corresponding authors:**

- William J. Jusko, Ph.D. ORCID: 0000-0003-4027-0550

Division of Pharmacokinetics, Pharmacodynamics, and Systems Pharmacology, Department of Pharmaceutical Sciences, School of Pharmacy and Pharmaceutical Sciences, State University of New York at Buffalo, 404 Pharmacy Building, Buffalo, NY14214-803, USA. Email: [wjjusko@buffalo.edu](mailto:wjjusko@buffalo.edu)

- Yoo-Seong Jeong, Ph.D.

Email: [j.yooseong@gmail.com](mailto:j.yooseong@gmail.com)

**Supplemental Table S1A.** Literature sources and reported values for blood volumes

| **Species** | **Reported Value** | **Reference** |
| --- | --- | --- |
| Duck | 86.3 (mL/kg) | (Portman et al., 1952) |
| Rabbit | 152.7 (mL) | (Baby et al., 2014) |
| Chicken | 10%∙$BW$ | (Newell & Shaffner, 1950) |
| Goat | 70 (mL/kg) | (Courtice, 1943) |
| Dog | 84 (mL/kg) | (Gibson 2nd et al., 1938) |
| Sheep | 59 (mL/kg) | (Hansard, 1956) |
| Pig | 60 (mL/kg) | (Bush et al., 1955) |
| Human | 5 (L) | (Sharma & Sharma, 2018) |

**Supplemental Table S1B.** Literature sources and reported values for hepatic blood flow

| **Species** | **Reported Value**  **(L/min)** | **Calculated** $\boldsymbol{F}^{\boldsymbol{*}}=1-CL_{H}/(Q_{H}\cdot R_{b})$ | **Reference** |
| --- | --- | --- | --- |
| Rabbit | 0.180 | 0.81 | (Balabaud et al., 1975) |
| Goat | 0.48 | 0.63 | (Boxenbaum, 1980) |
| Dog | 0.676 | 0.72 | (Boxenbaum, 1980) |
| Humans | 1.5 | 0.80 | (Rocha, 2012) |
| Sheep | 2.43 | 0.76 | (Boxenbaum, 1980) |
| Pig | 3.36 | 0.92 | (Boxenbaum, 1980) |

**Supplemental Table S1C.** Literature sources and reported values for cardiac output

| **Species** | **Reported**  **Body Weight (kg)** | **Calculated**  **Cardiac Output (L/h)^a^** | **Reference** |
| --- | --- | --- | --- |
| Duck | 2.5 | 28.0 | (Tabari et al., 2022) |
| Rabbit | 3 | 32.1 | (García et al., 1992) |
| Chicken | 4.5 | 43.6 | (El-Kholy et al., 2006) |
| Goat | 18 | 123.2 | (Nielsen & Rasmussen, 1983) |
| Dog | 20.7 | 136.8 | (Watson et al., 1988) |
| Sheep | 26 | 162.4 | (Fernandez et al., 1997) |
| Pig | 39.2 | 220.9 | (Galtier et al., 1983) |
| Human | 70 | 341.2 | (Luyckx et al., 1982) |

^a^Calculated by equation $Q_{co} \left( L/min \right)=0.235\cdot BW\left( kg \right)^{0.75}$

**Supplemental Table S2.** Literature sources and PK parameters for various non-human species after IV LVM dosing.

| **Reference** | **Species** | **Weight**  **(kg)** | **Dosing**  **Route** | **Dose**  **(mg/kg)** | $\boldsymbol{AUC}_{\mathbf{0}}^{\mathbf{t}}$  **(mg**$\boldsymbol{\cdot}$**h/L)** | $\boldsymbol{AUC}_{\mathbf{0}}^{\boldsymbol{\infty}}$  **(mg**$\boldsymbol{\cdot}$**h/L)** | $\boldsymbol{MRT}$**(h)** | $\boldsymbol{T}_{\mathbf{1/2}}$**(h)** | **Reported** $\boldsymbol{CL}$ **(L/h/kg)** | **Reported** $\boldsymbol{V}_{\boldsymbol{ss}}$ **(L/kg)** | **NCA**  $\boldsymbol{CL}$ **(L/h/kg)** | **NCA**  $\boldsymbol{V}_{\boldsymbol{ss}}$ **(L/kg)** |
| --- | --- | --- | --- | --- | --- | --- | --- | --- | --- | --- | --- | --- |
| **(Corum et al., 2019)** | **Red‐eared slider turtles** | 0.52 | IV | 10 | - | 75.38 | 5.95 | 5.00 | 0.14 | 0.81 | - | - |
| **(Youssefi et al., 2023)** | **Caspian Turtle** | 1 | IV | 10 | 44.81 | 46.21 | 5.59 | 5.16 | 0.22 | 1.06 | - | - |
| **(Sadati et al., 2021)** | **Belugas (Husohuso)** | 1 | IV | 50 | 156.7 | 168.55 | 6.45 | 8.99 | 0.31 | 1.32 | - | - |
| **(Sadati et al., 2021)** | **Belugas (Husohuso)** | 1 | IV | 100 | 275.8 | 298.73 | 6.61 | 10.1 | 0.363 | 1.43 | - | - |
| **(Tabari et al., 2022)** | **Duck ^b^** | 2.5 | IV | 30 | 109.1 | 115.76 | 7.08 | 6.60 | 0.28 | 1.51 | 0.24 | 1.82 |
| **(Villanueva et al., 2003)** | **Rabbit ^b^** | 2.5-3.5 | IV | 12.5 | 4.16 | - | 1.37 | 1.30 | 2.94 | 4.26 |  |  |
| **(Villanueva et al., 2003)** | **Rabbit ^b^** | 2.5-3.5 | IV | 16.0 | 6.18 | - | 1.49 | 1.42 | 2.63 | 4.33 | 2.55 | 4.23 |
| **(Villanueva et al., 2003)** | **Rabbit ^b^** | 2.5-3.5 | IV | 20.0 | 8.49 | - | 1.27 | 1.16 | 2.36 | 3.20 | - | - |
| **(García et al., 1992)** | **Rabbit** | 2.5-3.1 | IV | 12.5 | 4.07 | - | 1.15 | 0.99 | 3.21 | 3.76 | - | - |
| **(García et al., 1992)** | **Rabbit** | 2.5-3.1 | IV | 16.0 | 6.04 | - | 1.19 | 0.98 | 2.55 | 3.02 | - | - |
| **(García et al., 1992)** | **Rabbit** | 2.5-3.1 | IV | 20.0 | 8.68 | - | 1.22 | 0.96 | 2.53 | 2.94 | - | - |
| **(Pereda et al., 2002)** | **Rabbit** | 2.5-3.4 | IV | 12.5 | - | 3.42 | 1.21 | 0.63 | 3.04 | 3.13 | 3.70 | 4.24 |
| **(Pereda et al., 2002)** | **Rabbit** | 2.5-3.4 | IV | 16.0 | - | 5.37 | 1.14 | 0.77 | 2.60 | 2.77 | 3.05 | 3.47 |
| **(Pereda et al., 2002)** | **Rabbit** | 2.5-3.4 | IV | 20.0 | - | 7.19 | 1.19 | 0.68 | 2.31 | 2.49 | 4.32 | 5.17 |
| **(El-Kholy et al., 2006)** | **Chicken ^b^** | 4.5 | IV | 40 | - | 16 | 3.44 | 5.70 | 2.46 | 8.36 | 2.36 | 8.07 |
| **(Sahagún et al., 2000)** | **Goat** | 15-21 | IV | 7.5 | 1.89 | - | 0.71 | 1.32 | 0.58 | 2.97 | 0.36 | 2.97 |
| **(Nielsen & Rasmussen, 1983)** | **Goat ^b^** | 18 ^a^ | IV | 5 | - | 2.14 | 1.00 | 2.38 | 0.64 | 3.1 | 3.5 | 3.51 |
| **(Watson et al., 1988)** | **Dog ^b^** | 20.7 | IV | 10 | - | 18.06 | 2.73 | 1.79 | 0.54 | 1.42 | 0.55 | 1.69 |
| **(Fernandez et al., 1997)** | **Sheep ^b^** | 22-30 | IV | 5 | 4.25 | - | 1.81 | 1.48 | 1.16 | 2.14 | - | - |
| **(Fernandez et al., 1997)** | **Sheep ^b^** | 22-30 | IV | 7.5 | 5.14 | - | 1.56 | 1.34 | 1.54 | 2.39 | - | - |
| **(Fernandez et al., 1997)** | **Sheep ^b^** | 22-30 | IV | 10 | 10.11 | - | 2.13 | 1.72 | 1.01 | 2.14 | 0.95 | 2.07 |
| **(Galtier et al., 1983)** | **Pig ^b^** | 39.2 | IV | 5 | 12.9 | - | 11.38 | 9.45 | 0.39 | - | 0.42 | 4.73 |

^a^Values from the same species literature.

^b^Data used for 2CM and mPBPK modeling.

**Supplemental Table S3.** Literature sources and PK parameters of LVM in non-human species after extravascular dosing.

| **Reference** | **Species** | **Weight**  **(kg)** | **Dosing**  **Route** | **Dose**  **(mg/kg)** | $\boldsymbol{AUC}_{\boldsymbol{0}}^{\boldsymbol{t}}$  **(mg**$\boldsymbol{\cdot}$**h/L)** | $\boldsymbol{AUC}_{\mathbf{0}}^{\boldsymbol{\infty}}$  **(mg**$\boldsymbol{\cdot}$**h/L)** | $\boldsymbol{MRT}$ **(h)** | $\boldsymbol{k}_{\boldsymbol{a}}$**^c^ (1/h)** | $\boldsymbol{T}_{\boldsymbol{1/2}}$ **(h)** | $\boldsymbol{C}_{\boldsymbol{max}}$  **(μg/mL)** | $\boldsymbol{T}_{\boldsymbol{max}}$  **(h)** | $\boldsymbol{F}$ **(%)** |
| --- | --- | --- | --- | --- | --- | --- | --- | --- | --- | --- | --- | --- |
| **(Zanon et al., 2013)** | **Speckled Surubim** | 0.39 | IM | 50 | 17.85 | 18.31 | - | - | 1.86 | 12.79 | 0.5 | - |
| **(Tabari et al., 2020)** | **Caspian salmon** | 0.5 | IM | 25 (mg) | 44.62 | 49.97 | 5.71 | - | 4.56 | 35.53 | 0.25 | - |
| **(Corum et al., 2019)** | **Red‐eared slider turtles** | 0.52 | IM | 10 | - | 69.33 | 9.74 | - | 7.88 | 9.63 | 0.5 | 93.0 |
| **(Corum et al., 2019)** | **Red‐eared slider turtles** | 0.52 | SC | 10 | - | 84.93 | 11.85 | - | 9.43 | 10.51 | 0.5 | 115.3 |
| **(Youssefi et al., 2023)** | **Caspian Turtle** | 1 | SC | 10 | 70.26 | 82.69 | 12.03 | 0.16 | 9.16 | 10.53 | 0.5 | 178.9 |
| **(Sadati et al., 2021)** | **Belugas (Huso-huso)** | 1 | PO | 100 | 63.35 | 72.81 | 9.96 | 0.28 | 11.51 | 18.63 | 1 | 43.2 |
| **(Sadati et al., 2021)** | **Belugas (Huso-huso)** | 1 | PO | 100 | 131.16 | 148.23 | 9.53 | 0.34 | 8.42 | 38.98 | 1 | 49.6 |
| **(Graziani & De Martin, 1977b)** | **Rat** | 0.68 | PO | 100 | - | 21.14 ^a^ | 5.73 ^b^ | - | 5.03 ^a^ | 8.85 | 1 | - |
| **(Graziani & De Martin, 1977b)** | **Rat** | 0.46 | PO | 100 | - | 28.56 ^a^ | 7.97 ^b^ | - | 7.84 ^a^ | 9.9 | 0.5 | - |
| **(Tabari et al., 2022)** | **Duck** ^b^ | 2.5 | PO | 30 | 50.45 | 50.94 | 4.59 | - | 4.18 | 10.42 | 1.75 | 46.3 |
| **(García et al., 1992)** | **Rabbit** ^b^ | 2.1-3.3 | PO | 12.5 | - | 2.11 | 3.26 | 0.47 | - | 0.71 | 0.77 | 52.9 |
| **(García et al., 1992)** | **Rabbit** ^b^ | 2.1-3.3 | PO | 16.0 | - | 3.94 | 2.83 | 0.60 | 3.74 | 1.32 | 1.6 | 62.0 |
| **(García et al., 1994)** | **Rabbit** ^b^ | 2.1-3.3 | PO | 20.0 | - | 7.00 | 5.43 | 0.24 | - | 1.77 | 1.4 | 80.7 |
| **(El-Kholy et al., 2006)** | **Chicken** ^b^ | 4.5 | PO | 40 | - | 9.98 | 3.9 | 2.17 | 6.06 | 2.92 | 1 | 61 |
| **(Sahagún et al., 2001)** | **Goat** ^b^ | 15-21 | PO | 7.5 | 1.24 | 1.28 | 1.50 | 1.27 | 1.31 | 0.74 | 0.56 | 66.4 |
| **(Sahagún et al., 2000)** | **Goat** | 15-21 | SC | 7.5 | 1.46 | - | 1.20 | 2.04 | 0.52 | 1.11 | 0.30 | 73.1 |
| **(Gokbulut et al., 2014)** | **Goat** | 23.1 | PO | 7.5 | 2.09 | - | 2.69 | - | 3.81 | 1.07 | 0.83 | - |
| **(Galtier et al., 1981)** | **Goat** | 31.5 | PO | 9.97 | - | 1.67 ^d^ | - | - | 0.68 | 0.63 | 0.17 | - |

**Supplemental Table S3. continued**

| **Reference** | **Species** | **Weight**  **(kg)** | **Dosing**  **Route** | **Dose**  **(mg/kg)** | $\boldsymbol{AUC}_{\boldsymbol{0}}^{\boldsymbol{t}}$  **(mg**$\boldsymbol{\cdot}$**h/L)** | $\boldsymbol{AUC}_{\mathbf{0}}^{\boldsymbol{\infty}}$  **(mg**$\boldsymbol{\cdot}$**h/L)** | $\boldsymbol{MRT}$ **(h)** | $\boldsymbol{k}_{\boldsymbol{a}}$ **^c^ (1/h)** | $\boldsymbol{T}_{\mathbf{1/2}}$ **(h)** | $\boldsymbol{C}_{\boldsymbol{max}}$  **(μg/mL)** | $\boldsymbol{T}_{\boldsymbol{max}}$  **(h)** | $\boldsymbol{F}$ **(%)** |
| --- | --- | --- | --- | --- | --- | --- | --- | --- | --- | --- | --- | --- |
| **(Galtier et al., 1981)** | **Goat** | 31.0 | IM | 7.51 | - | 1.62 ^d^ | - | - | 0.50 | 1.26 | 0.25 | - |
| **(Galtier et al., 1981)** | **Goat** | 29.0 | SC | 7.5 | - | 1.77 ^d^ | - | - | 0.76 | 0.54 | 0.75 | - |
| **(Watson et al., 1988)** | **Dog** | 20.7 | PO | 10 | - | 12.18 | 3.43 | 1.43 | 1.28 | 3.33 | 1.8 | 64 |
| **(Luque et al., 2021)** | **Lamb** | 26.6 | SC | 8 | 7.41 | 7.65 | - | - | 3.2 | 2.04 | 1.46 | - |
| **(McKellar et al., 1991)** | **Lamb** | 13.9 | PO | 7.5 | - | 5.99 | 5.90 | - | 3.14 | 0.83 | 2 | - |
| **(McKellar et al., 1991)** | **Lamb** | 13.9 | SC | 7.5 | - | 3.89 | 3.59 | - | 3.28 | 1.19 | 0.5 | - |
| **(Fernandez et al., 1998)** | **Sheep** | 22-30 | IM | 5.0 | 3.35 | - | - | - | 2.05 | 1.61 | 0.42 | 77.7 |
| **(Fernandez et al., 1998)** | **Sheep** | 22-30 | IM | 7.5 | 3.90 | - | - | - | 2.14 | 2.15 | 0.38 | 75.6 |
| **(Fernandez et al., 1998)** | **Sheep** | 22-30 | IM | 10 | 8.36 | - | - | - | 2.34 | 3.43 | 0.41 | 82.8 |
| **(Fernandez et al., 1998)** | **Sheep** ^b^ | 22-30 | PO | 5.0 | 2.67 | - | - | - | 4.05 | 0.80 | 0.66 | 61.1 |
| **(Fernandez et al., 1998)** | **Sheep** ^b^ | 22-30 | PO | 7.5 | 3.13 | - | 1.7 | 7.14 | 2.77 | 1.13 | 0.71 | 60.4 |
| **(Fernandez et al., 1998)** | **Sheep** ^b^ | 22-30 | PO | 10 | 6.73 | - | - | - | 5.44 | 1.65 | 0.80 | 66.6 |
| **(Graziani & De Martin, 1977b)** | **Calf** | 34.88 | PO | 10 | - | 7.34 | 1.80 | - | 1.33 | 5.9 | 0.5 | - |
| **(Graziani & De Martin, 1977b)** | **Calf** | 34.88 | SC | 10 | - | 17.18 | 1.80 | - | 0.89 | 9.5 | 0.5 | - |
| **(Galtier et al., 1983)** | **Pig** | 41.4 | IM | 7.5 | 17.97 | - | - | - | 6.38 | 1.82 |  | 83 |
| **(Galtier et al., 1983)** | **Pig** ^b^ | 38.3 | PO | 10 | 13.08 | - | 11.68^a^ | 3.33 | 6.56 | 1.34 | 1.46 ^a^ | 62 |
| **(Graziani & De Martin, 1977b)** | **Bull** | 111-211 (Mean 168) | IM | 8 | - | 8.71 ^a^ | 4.14 ^a^ | - | 3.66 ^a^ | >3 | 0.5 | - |
| **(Ho et al., 2009)** | **Horse** | 547 | SC | 4.2 | - | 2.54 | 4.04 | - | 2.36 | 0.614 | 1.44 | - |

**Supplemental Table S3. continued**

| **Reference** | **Species** | **Weight**  **(kg)** | **Dosing**  **Route** | **Dose**  **(mg/kg)** | $\boldsymbol{AUC}_{\boldsymbol{0}}^{\boldsymbol{t}}$  **(mg**$\boldsymbol{\cdot}$**h/L)** | $\boldsymbol{AUC}_{\mathbf{0}}^{\boldsymbol{\infty}}$  **(mg**$\boldsymbol{\cdot}$**h/L)** | $\boldsymbol{MRT}$ **(h)** | $\boldsymbol{k}_{\boldsymbol{a}}$ **^c^ (1/h)** | $\boldsymbol{T}_{\mathbf{1/2}}$ **(h)** | $\boldsymbol{C}_{\boldsymbol{max}}$  **(μg/mL)** | $\boldsymbol{T}_{\boldsymbol{max}}$  **(h)** | $\boldsymbol{F}$ **(%)** |
| --- | --- | --- | --- | --- | --- | --- | --- | --- | --- | --- | --- | --- |
| **(Ho et al., 2009)** | **Horse** | 547 | PO | 5.1 | - | 1.47 | 4.28 | - | 2.05 | 0.398 | 1.45 | - |
| **(Philip et al., 2023)** | **Horse** | 450 | PO | 5 | 14.48 | - | - | - | 18.95 | 0.81 | 0.75 | - |

^a^NCA using Phoenix WinNonlin^®^; ^b^Data used for 2CM and mPBPK modeling; ^c^Calculated using Equation (1). ^d^

**Supplemental Table S4.** Literature sources and PK parameters for LVM for oral (PO) dosing in humans.

| **Reference** | **Subjects No, sex** | **Weight (kg)** | **Type of subjects** | **Assay method**^a^ | **Dosing**  **Route** | **Dose**  **(mg/kg)** | $\boldsymbol{CL}$  **(L/h/kg)** | $\boldsymbol{V}_{\boldsymbol{d}}$  **(L/kg)** |
| --- | --- | --- | --- | --- | --- | --- | --- | --- |
| **(Graziani & De Martin, 1977a)** | 3M | 73.67 | Healthy | GC | PO/tablet | 150 (mg) | CL/F = 0.5 | 3.06 |
| **(Graziani & De Martin, 1977a)** | 3M | 73.67 | Healthy | GC | PO/syrup | 150 (mg) | CL/F = 0.45 | 2.58 |
| **(Adams, 1978)** | 3M | 70 ^c^ | Healthy | GC | PO/syrup | 150 (mg) | CL/F = 0.77 | 8.76 |
| **(Luyckx et al., 1982)** | 6M 5W | 70 ^b, c^ | Healthy | GC | PO | 2.5 | 0.25 | 1.57 |
| **(Luyckx et al., 1982)** | 6M 5W | 70 ^b, c^ | Healthy | GC | PO | 5 | 0.28 | 1.41 |
| **(Luyckx et al., 1982)** | 6M 6W | 70 ^b^ | Cancer | GC | PO | 2.5 | 0.29 | 1.23 |
| **(Luyckx et al., 1982)** | 6M 6W | 70 ^b^ | Cancer | GC | PO | 5 | 0.25 | 1.31 |
| **(Kouassi et al., 1986)** | 7M 3W | 66 | Healthy | GC | PO | 150 (mg) | 0.51 | 3.80 |
| **(Gwilt et al., 2000)** | 9M 11W | 78.5 | Cancer | GC/MS | PO | 50 (mg) | 0.44 | 2.96 |
| **(Reid et al., 1998)** | 21M 17W | 70 ^b^ | Cancer | GC | PO | 100  (mg/m^2^) | 21.1 per m^2^ | - |

^a^GC = Gas chromatography; ^b^Values from the same species literature; ^c^Data used for 2CM and mPBPK modeling

**Supplemental Table S5.** Pharmacokinetic parameters (CV%) of the 2CM (Figure 2); individual fitting results for 8 species with intravenous (IV) dosing. (See Figure S1)

| **Parameter (Unit)** | **Duck** | **Rabbit** | **Chicken** | **Goat** | **Dog** | **Sheep** | **Pig** | **Human^a^** |
| --- | --- | --- | --- | --- | --- | --- | --- | --- |
| $\boldsymbol{V}_{\boldsymbol{p}}$ **(L)** | 1.514 (17.47) | 5.063 (8.38) | 12.22 (8.58) | 14.96 (1.68) | 6.236 (11.63) | 32.94 (7.61) | 87.71 (1.88) | 110.3 (2.67) |
| $\boldsymbol{V}_{\boldsymbol{t}}$ **(L)** | 2.813 (13.73) | 4.959 (9.24) | 24.06 (12.83) | 10.85 (4.75) | 1.192 (47.09) | 25.79 (11.56) | 97.09 (4.17) | 22.59 (23.42) |
| $\boldsymbol{C}\boldsymbol{l}_{\boldsymbol{d}}$ **(L/h)** | 2.095 (29.42) | 10.76 (17.98) | 4.93 (13.17) | 4.82 (5.83) | 0.568 (111.1) | 25.89 (22.36) | 61.76 (6.60) | 6.581 (40.06) |
| $\boldsymbol{CL}$ **(L/h)** | 0.615 (5.399) | 7.917 (2.44) | 10.94 (4.29) | 6.726 (0.93) | 2.357 (4.35) | 29.19 (2.529) | 16.41 (2.75) | 16.46 (2.50) |

^a^Human PK data were fitted with the bioavailability ($F$) fixed at 0.66 and the absorption rate constant ($k_{a}$) was fitted to be 2.65 h^-1^.

**Supplemental Table S6.** PK parameters (CV%) of the 2CM (Figure 2); individual fitting results for 7 species with intravenous (IV) and oral dosing. (See Figure S2)

| **Parameter (Unit)** | **Duck** | **Rabbit** | **Chicken** | **Goat** | **Dog** | **Sheep** | **Pig** | **Human^a^** |
| --- | --- | --- | --- | --- | --- | --- | --- | --- |
| $\boldsymbol{V}_{\boldsymbol{p}}$ **(L)** | 1.48 (8.64) | 4.74 (9.50) | 12.87 (13.29) | 14.0 (7.41) | 23.40 (27.96) | 34.61 (6.34) | 88.92 (5.06) | NA |
| $\boldsymbol{V}_{\boldsymbol{t}}$ **(L)** | 2.24 (11.62) | 5.45 (9.30) | 21.96 (16.62) | 13.10 (10.29) | 8.65 (66.18) | 33.29 (10.42) | 90.30 (7.44) | NA |
| $\boldsymbol{C}\boldsymbol{l}_{\boldsymbol{d}}$ **(L/h)** | 2.12 (18.63) | 11.37 (18.11) | 4.01 (18.96) | 5.98 (17.0) | 6.40 (136.8) | 22.88 (15.15) | 56.68 (13.89) | NA |
| $\boldsymbol{CL}$ **(L/h)** | 0.652 (5.17) | 8.02 (3.00) | 10.90 (6.91) | 6.65 (2.58) | 11.60 (8.09) | 28.35 (2.91) | 17.22 (3.45) | NA |
| $\boldsymbol{k}_{\boldsymbol{a}}$ **(h^-1^)** | 0.80 (42.42) | 0.50 (6.45) | 0.84 (14.54) | 3.56 (57.6) | 1.294 (171.1) | 1.585 (8.11) | 1.162 (7.67) | NA |
| $\boldsymbol{F}$ | 0.425 (26.09) | 0.774 (5.26) | 0.52 (9.82) | 0.11 (6.96) | 0.65 (11.76) | 0.54 (4.03) | 0.58 (3.31) | NA |

^a^IV data were not available (NA).

**Supplemental Table S7.** Pharmacokinetic parameters of the mPBPK model (Figure 3) individual fitting results for 8 species with intravenous (IV) dosing. (See Figure S3)

| **Parameter (unit)** | **Duck** | **Rabbit** | **Chicken** | **Goat** | **Dog** | **Sheep** | **Pig** | **Human^b^** |
| --- | --- | --- | --- | --- | --- | --- | --- | --- |
| $\boldsymbol{K}_{\boldsymbol{p}}$ | 1.53 (8.33) | 3.00 (4.41) | 6.01 (6.06) | 1.38 (1.59) | 1.55 (29.57) | 1.51 (4.95) | 4.46 (2.68) | 1.88 (58.42) |
| $\boldsymbol{f}_{\boldsymbol{d}\boldsymbol{1}}$ | 0.073 (16.42) | 0.281 (7.57) | 0.078 (10.83) | 0.034 (6.05) | 0.001 (1244) | 0.101 (23.55) | 0.23 (8.01) | 0.059 (4.51) |
| $\boldsymbol{f}_{\boldsymbol{d}\boldsymbol{2}}$^a^ | 0.927 | 0.719 | 0.922 | 0.966 | 0.999 | 0.899 | 0.77 | 0.941 |
| $\boldsymbol{f}_{\boldsymbol{t}}$ | 0.69 (4.83) | 0.75 (2.04) | 0.72 (4.02) | 0.48 (2.44) | 0.023 (879) | 0.54 (9.44) | 0.68 (2.68) | 0.40 (32.68) |
| $\boldsymbol{CL}$ **(L/h)** | 0.632 (5.89) | 6.729 (1.80) | 9.12 (3.41) | 6.38 (0.71) | 10.70 (6.91) | 25.99 (2.40) | 15.39 (1.19) | 16.86 (2.50) |

^a^The value of $f_{d2}$ is calculated as $f_{d, total}-f_{d1}$, with the assumption of $f_{d,total}=1$

^b^Human PK data were fitted with the bioavailability ($F$) fixed at 0.66, and the absorption rate constant ($k_{a}$) was fitted to be 1.156 h^-1^.

**Supplemental Table S8.** Pharmacokinetic parameters (CV%) of the mPBPK model (Figure 3); individual fitting results for 7 species with intravenous (IV) and oral data. (See Figure S4)

| **Parameter (unit)** | **Duck** | **Rabbit** | **Chicken** | **Goat** | **Dog** | **Sheep** | **Pig** | **Human^b^** |
| --- | --- | --- | --- | --- | --- | --- | --- | --- |
| $\boldsymbol{K}_{\boldsymbol{p}}$ | 1.70 (8.23) | 3.17 (2.86) | 5.96 (8.79) | 1.35 (2.42) | 1.48 (12.55) | 3.47 (3.47) | 4.49 (3.45) | NA |
| $\boldsymbol{f}_{\boldsymbol{d}\boldsymbol{1}}$ | 0.064 (30.79) | 0.263 (6.40) | 0.075 (11.85) | 0.037 (5.93) | 0.035 (248.8) | 0.139 (12.29) | 0.215 (10.47) | NA |
| $\boldsymbol{f}_{\boldsymbol{d}\boldsymbol{2}}$**^a^** | 0.936 | 0.737 | 0.925 | 0.963 | 0.965 | 0.861 | 0.785 | NA |
| $\boldsymbol{f}_{\boldsymbol{t}}$ | 0.695 (9.36) | 0.742 (2.06) | 0.715 (3.97) | 0.48 (2.60) | 0.24 (126.6) | 0.60 (4.82) | 0.667 (3.11) | NA |
| $\boldsymbol{CL}$ **(L/h)** | 0.61 (5.33) | 6.65 (1.09) | 9.10 (3.67) | 6.42 (0.92) | 11.17 (8.25) | 25.53 (1.53) | 15.43 (3.12) | NA |
| $\boldsymbol{F}$ | 0.38 (26.51) | 0.437 (13.89) | 0.421 (12.42) | 0.111 (95.59) | 0.608 (12.36) | 0.469 (4.73) | 0.532 (7.36) | NA |
| $\boldsymbol{k}_{\boldsymbol{a}}$**(h^-1^)** | 0.776 (41.95) | 0.451 (14.85) | 0.729 (17.12) | 3.19 (11.73) | 1.37 (733.8) | 0.931 (7.63) | 0.556 (13.64) | NA |

^a^The value of $f_{d2}$ was calculated as $f_{d,total}-f_{d1}$, with the assumption of $f_{d,total}=1$

^b^IV data were not available (NA).

**Supplemental Table S9.** Bioavailability ($F$) values for levamisole

| **Species** | **Reported Value of** $\boldsymbol{F}$ | **Reference** |
| --- | --- | --- |
| Duck | 46.3% | (Tabari et al., 2022) |
| Beluga (huso-huso) | 43.2% | (Sadati et al., 2021) |
| Caspian salmon | 69.3% | (Tabari et al., 2020) |
| Chickens (pre-lay) | 61% | (El-Kholy et al., 2006) |
| Chickens (peak production) | 88% | (El-Kholy et al., 2006) |
| Rabbits | 53.0% to 80.7% | (García et al., 1994) |
| Goats | 66.44% | (Galtier et al., 1981) |
| Dogs | 64% | (Watson et al., 1988) |
| Sheep | 60.41% - 66.60% | (Fernandez et al., 1998) |
| Pigs | 62% | (Galtier et al., 1983) |
| Human | 62.5% to 68% | (Kouassi et al., 1986; Luyckx et al., 1982) |

**Supplemental Table S10.** Clearance ($CL$, L/h/kg) values for levamisole

| **Species** | **Reported Value (L/h/kg)** | **Reference** |
| --- | --- | --- |
| Red-eared slider turtle | 0.14 | (Corum et al., 2019) |
| Caspian turtle | 0.22 | (Youssefi et al., 2023) |
| Beluga (huso-huso) | 0.31 | (Sadati et al., 2021) |
| Ducks | 0.27 | (García et al., 1992) |
| Chickens (prelay) | 2.46 | (El-Kholy et al., 2006) |
| Human (healthy and cancer) | 0.25 to 0.51 | (Kouassi et al., 1986; Luyckx et al., 1982) |

**
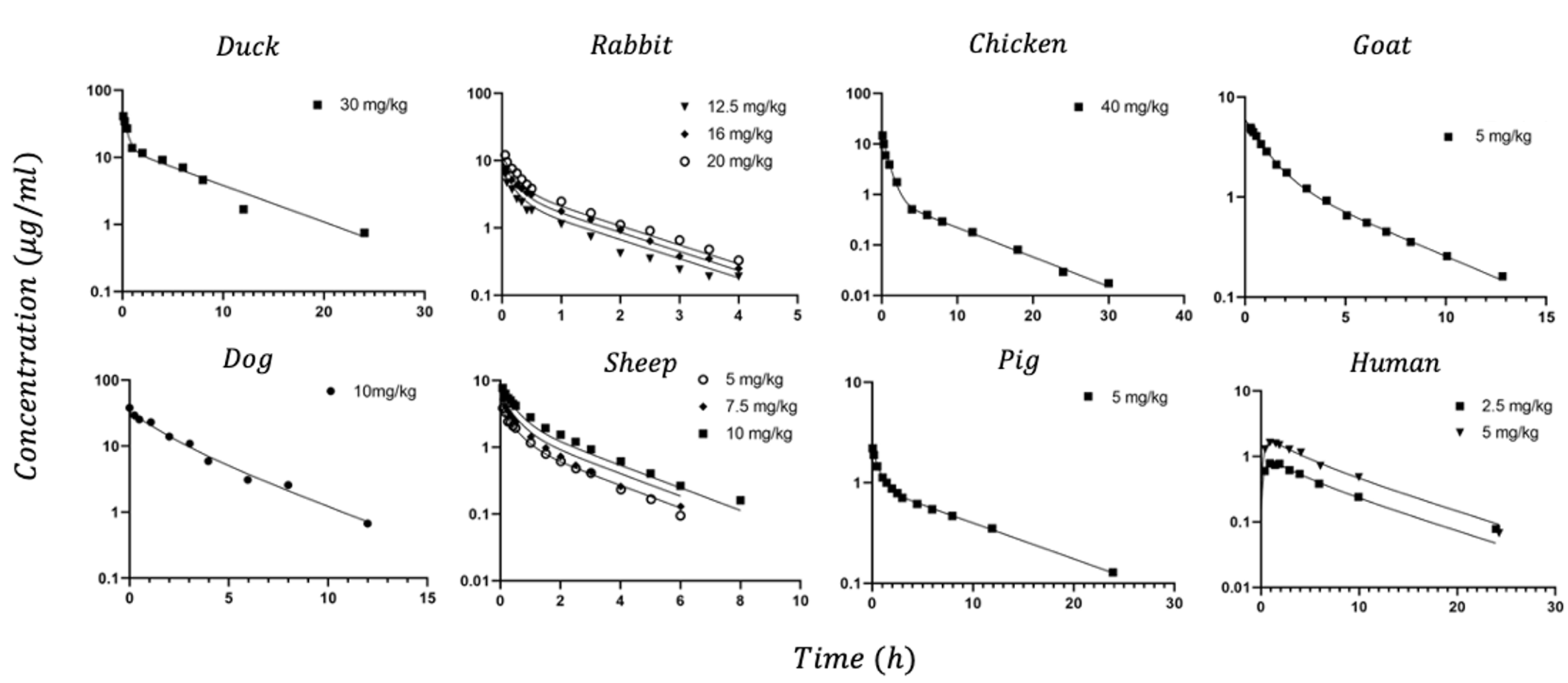
**

**Supplemental Figure S1.** Two-compartment model individual fittings for 8 species with IV dosing.

Parameter estimates are listed in Table S5.

**
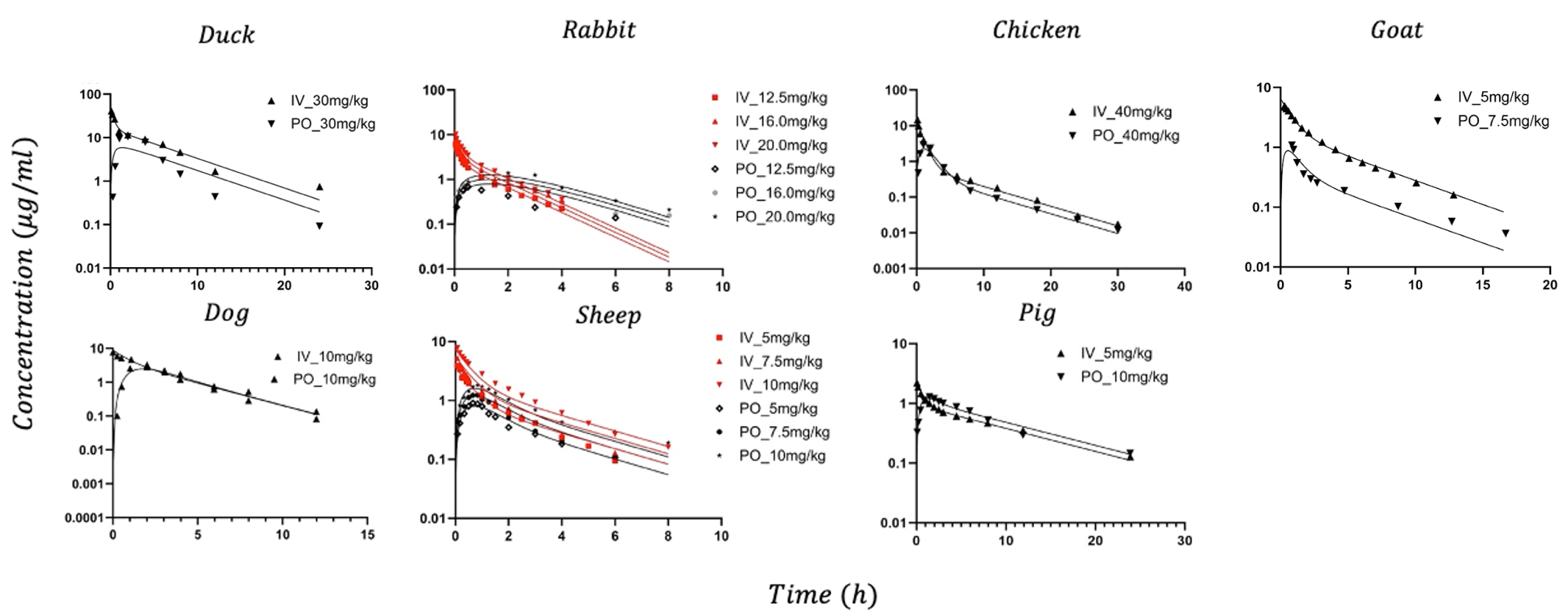
**

**Supplemental Figure S2.** Two-compartment model with simultaneous oral and IV data fittings jointly for 7 species.

Parameter estimates are listed in Table S6.

**
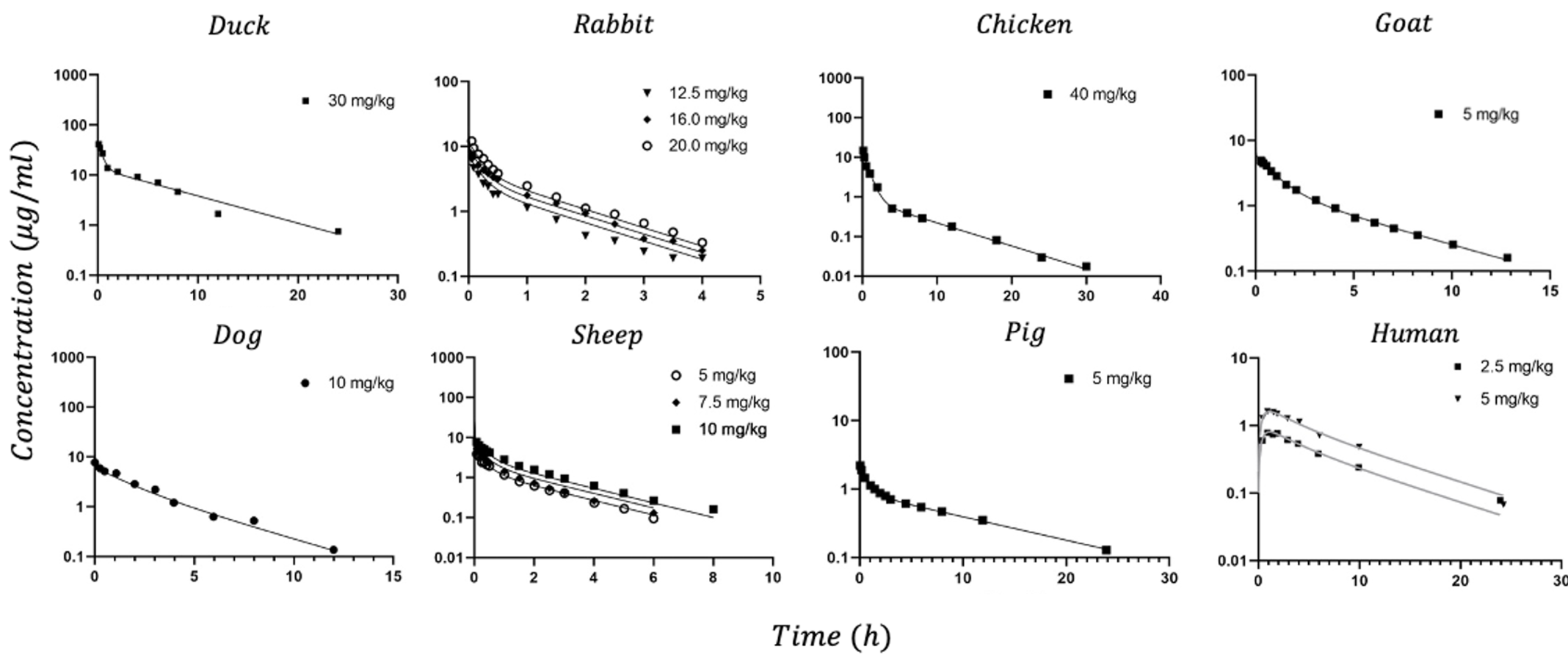
**

**Supplemental Figure S3.** Minimal physiologically based pharmacokinetic (mPBPK) model individual fittings for 8 species with IV dosing. Parameter estimates are listed in Table S7.


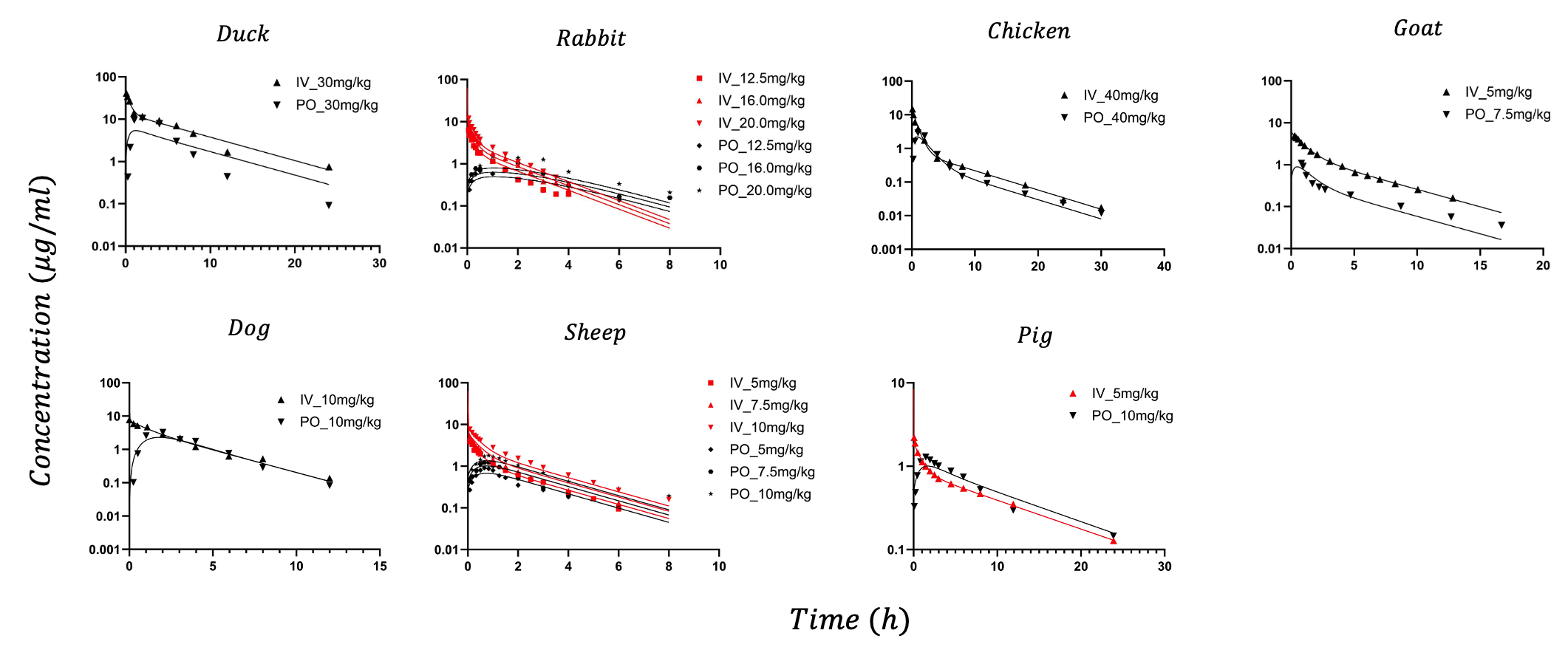


**Supplemental Figure S4.** Minimal physiologically based pharmacokinetic model with joint Oral and IV data fitting for 7 species.

Parameter estimates are listed in Table S8.

**Supplemental Figure S5.** Comparison of clearance values obtained from various sources and methods. All clearance values are listed in Table 1.

**REFERENCES**

Adams, J. (1978). Pharmacokinetics of Levamisole. *JRheumatol*.

Baby, P. M., Kumar, P., Kumar, R., Jacob, S. S., Rawat, D., Binu, V., & Karun, K. M. (2014). A novel method for blood volume estimation using trivalent chromium in rabbit models. *Indian Journal of Plastic Surgery*, *47*(02), 242-248.

Balabaud, C., Roche, M., & Dangoumau, J. (1975). Measurement of hepatic blood flow in the unanesthetized rabbit using 198Au and 125I Rose Bengal clearance technique. *Biomedicine/[publiee Pour l'AAICIG]*, *23*(9), 353-355.

Boxenbaum, H. (1980). Interspecies variation in liver weight, hepatic blood flow, and antipyrine intrinsic clearance: extrapolation of data to benzodiazepines and phenytoin. *Journal of pharmacokinetics and biopharmaceutics*, *8*(2), 165-176.

Bush, J., Jensen, W., Cartwright, G., & Wintrobe, M. (1955). Blood volume studies in normal and anemic swine. *American Journal of Physiology-Legacy Content*, *181*(1), 9-14.

Corum, O., Durna Corum, D., Atik, O., Altan, F., Er, A., & Uney, K. (2019). Pharmacokinetics of levamisole in the red-eared slider turtles (Trachemys scripta elegans). *J Vet Pharmacol Ther*, *42*(6), 654-659. <https://doi.org/10.1111/jvp.12763>

Courtice, F. (1943). The blood volume of normal animals. *The Journal of physiology*, *102*(3), 290.

El-Kholy, H., Kemppainen, B., Ravis, W., & Hoerr, F. (2006). Pharmacokinetics of levamisole in broiler breeder chickens. *J Vet Pharmacol Ther*, *29*(1), 49-53. <https://doi.org/10.1111/j.1365-2885.2006.00710.x>

Fernandez, M., García, J. J., Sierra, M., Diez, M., & Terán, M. (1997). Pharmacokinetics of levamisole in sheep after intravenous administration. *New Zealand Veterinary Journal*, *45*(2), 63-66.

Fernandez, M., García, J. J., Sierra, M., Diez, M., & Teran, M. (1998). Bioavailability of levamisole after intramuscular and oral administration in sheep. *New Zealand Veterinary Journal*, *46*(5), 173-176.

Galtier, P., Escoula, L., & Alvinerie, M. (1983). Pharmacokinetics of [3H]levamisole in pigs after oral and intramuscular administration. *Am J Vet Res*, *44*(4), 583-587.

Galtier, P., Escoula, L., Camguilhem, R., & Alvinerie, M. (1981). Comparative bioavailability of levamisole in non lactating ewes and goats. Annales de Recherches Veterinaires,

García, J. J., Diez, M., Sierra, M., & Terán, M. (1992). Pharmacokinetics of levamisole in rabbits after intravenous administration. *Journal of Veterinary Pharmacology Therapeutics*, *15*(1), 85-90.

García, J. J., Diez, M., Sierra, M., & Terán, M. (1994). Bioavailability of levamisole administered by subcutaneous and oral routes in rabbits. *Journal of Veterinary Pharmacology and Therapeutics*, *17*(2), 135-140.

Gibson 2nd, J. G., Keeley, J. L., & Pijoan, M. (1938). The blood volume of normal dogs. *American Journal of Physiology-Legacy Content*, *121*(3), 800-806.

Gokbulut, C., Yalinkilinc, H. S., Aksit, D., & Veneziano, V. (2014). Comparative pharmacokinetics of levamisole-oxyclozanide combination in sheep and goats following per os administration. *Canadian Journal of Veterinary Research*, *78*(4), 316-320.

Graziani, G., & De Martin, G. (1977a). Pharmacokinetic studies on levamisole: on the pharmacokinetics and relative bioavailability of levamisole in man. *Drugs Exp Clin Res*, *2*, 235-240.

Graziani, G., & De Martin, G. (1977b). Pharmacokinetic studies on levamisole. Absorption, distribution, excretion and metabolism of levamisole in animals. A review. *Drugs exp. clin. Res*, *2*, 221.

Gwilt, P., Tempero, M., Kremer, A., Connolly, M., & Ding, C. (2000). Pharmacokinetics of levamisole in cancer patients treated with 5-fluorouracil. *Cancer chemotherapy and pharmacology*, *45*, 247-251.

Hansard, S. L. (1956). Residual organ blood volume of cattle, sheep and swine. *Proceedings of the Society for Experimental Biology and Medicine*, *91*(1), 31-34.

Ho, E. N., Leung, D. K., Leung, G. N., Wan, T. S., Wong, A. S., Wong, C. H., Soma, L. R., Rudy, J. A., Uboh, C., & Sams, R. (2009). Aminorex and rexamino as metabolites of levamisole in the horse. *Anal Chim Acta*, *638*(1), 58-68. <https://doi.org/10.1016/j.aca.2009.02.033>

Kouassi, E., Caillé, G., Léry, L., Larivière, L., & Vézina, M. (1986). Novel assay and pharmacokinetics of levamisole and p-hydroxylevamisole in human plasma and urine. *Biopharm Drug Dispos*, *7*(1), 71-89. <https://doi.org/10.1002/bdd.2510070110>

Luque, S., Lloberas, M., Cardozo, P., Virkel, G., Farias, C., Viviani, P., Lanusse, C., Alvarez, L., & Lifschitz, A. (2021). Combined moxidectin-levamisole treatment against multidrug-resistant gastrointestinal nematodes: A four-year efficacy monitoring in lambs. *Vet Parasitol*, *290*, 109362. <https://doi.org/10.1016/j.vetpar.2021.109362>

Luyckx, M., Rousseau, F., Cazin, M., Brunet, C., Cazin, J., Haguenoer, J., Devulder, B., Lesieur, I., Lesieur, D., & Gosselin, P. (1982). Pharmacokinetics of levamisole in healthy subjects and cancer patients. *European journal of drug metabolism and pharmacokinetics*, *7*, 247-254.

McKellar, Q., Jackson, F., Coop, R., Jackson, E., & Scott, E. (1991). Effect of parasitism with Nematodirus battus on the pharmacokinetics of levamisole, ivernectin and netobimin. *Veterinary Parasitology*, *39*(1-2), 123-136.

Newell, G. W., & Shaffner, C. S. (1950). Blood Volume Determinations in Chickens. *Poultry Science*, *29*(1), 78-87. <https://doi.org/10.3382/ps.0290078>

Nielsen, P., & Rasmussen, F. (1983). Pharmacokinetics of levamisole in goats and pigs. In *VETERINARY PHARMACOLOGY AND TOXICOLOGY* (pp. 241-244). Springer.

Pereda, P., Garcia, J. J., Sierra, M., Fernandez, N., Sahagun, A. M., & Diez, M. J. (2002). Intra-arterial pharmacokinetics and pulmonary first-pass of levamisole in rabbits. *Pharmacol Res*, *45*(4), 285-289. <https://doi.org/10.1006/phrs.2002.0966>

Philip, M., Karakka Kal, A. K., Subhahar, M. B., Karatt, T. K., Mathew, B., Perwad, Z., Graiban, F. M., Caveney, M. R., Sayed, R., & Kadry, A. M. (2023). Hydroxy levamisole and its phase II conjugates as potential indicators of levamisole doping in thoroughbred horses. *Rapid Commun Mass Spectrom*, *37*(3), e9430. <https://doi.org/10.1002/rcm.9430>

Portman, O. W., Mcconnell, K. P., & Rigdon, R. (1952). Blood volumes of ducks using human serum albumin labeled with radioiodine. *Proceedings of the Society for Experimental Biology and Medicine*, *81*(3), 599-601.

Reid, J. M., Kovach, J. S., O'Connell, M. J., Bagniewski, P. G., & Moertel, C. G. (1998). Clinical and pharmacokinetic studies of high-dose levamisole in combination with 5-fluorouracil in patients with advanced cancer. *Cancer chemotherapy and pharmacology*, *41*, 477-484.

Rocha, F. G. (2012). Chapter 4 - Liver blood flow: Physiology, measurement, and clinical relevance. In W. R. Jarnagin & L. H. Blumgart (Eds.), *Blumgart's Surgery of the Liver, Pancreas and Biliary Tract (Fifth Edition)* (pp. 74-86.e75). W.B. Saunders. <https://doi.org/https://doi.org/10.1016/B978-1-4377-1454-8.00004-7>

Sadati, N. Y., Youssefi, M. R., Hosseinifard, S. M., Tabari, M. A., & Giorgi, M. (2021). Pharmacokinetics and pharmacodynamics of single and multiple-dose levamisole in belugas (Huso huso): Main focus on immunity responses. *Fish Shellfish Immunol*, *114*, 152-160. <https://doi.org/10.1016/j.fsi.2021.04.016>

Sahagún, A. M., Garcia, J. J., Sierra, M., Fernandez, N., Diez, M. J., & Teran, M. T. (2000). Subcutaneous bioavailability of levamisole in goats. *J Vet Pharmacol Ther*, *23*(3), 189-192. <https://doi.org/10.1046/j.1365-2885.2000.00258.x>

Sahagún, A. M., Terán Somaza, M. T., García, J. J., Fernández, N., Sierra Vega, M., & Diez Liébana, M. J. (2001). Oral bioavailability of levamisole in goats.

Sharma, R., & Sharma, S. (2018). Physiology, blood volume.

Tabari, M. A., Pozniak, B., Mostafavi Niaki, S. T., Salehi, A., & Youssefi, M. R. (2022). Pharmacokinetics and therapeutic efficacy of levamisole in Ascaridia galli experimentally infected ducks. *Vet Parasitol*, *312*, 109838. <https://doi.org/10.1016/j.vetpar.2022.109838>

Tabari, M. A., Youssefi, M. R., Hosseinifard, S. M., Moghaddamnia, A. A., Kazemi, S., Sadati, N. Y., Jalali Mothahari, A., & Giorgi, M. (2020). Pharmacokinetics of levamisole after intramuscular and oral administrations to Caspian salmon (Salmo trutta caspius). *J Vet Pharmacol Ther*, *43*(3), 276-281. <https://doi.org/10.1111/jvp.12856>

Villanueva, I., Diez, M. J., García, J. J., Fernández, M. N., Sahagún, A. M., Sierra, A., & Sierra, M. (2003). Effect of first-pass hepatic metabolism on the disposition of levamisole after intravenous administration in rabbits. *American journal of veterinary research*, *64*(10), 1283-1287.

Watson, A., van Gogh, H., Sangster, N., & Church, D. (1988). Levamisole pharmacokinetics and bioavailability in dogs. *Research in veterinary science*, *45*(3), 411-413.

Youssefi, M. R., Khabbazian, F. G., Navidi, N., Yazdani Rostam, M. M., Giorgi, M., & Abouhosseini Tabari, M. (2023). Pharmacokinetics and therapeutic efficacies of fenbendazole in comparison with levamisole in helminth-infected Caspian turtles (Mauremys caspica). *J Vet Pharmacol Ther*, *46*(3), 170-176. <https://doi.org/10.1111/jvp.13094>

Zanon, R. B., Cerozi, B. S., Silva, T. S., & Cyrino, J. E. (2013). Pharmacokinetic of levamisole in speckled surubim Pseudoplatystoma corruscans. *J Vet Pharmacol Ther*, *36*(3), 298-301. <https://doi.org/10.1111/jvp.12002>
